# Amino acid metabolism of the thermophilic acetogen *Thermacetogenium phaeum*

**DOI:** 10.1371/journal.pone.0336914

**Published:** 2025-12-03

**Authors:** Yanlin Cai, Andreas Marquardt, David Schleheck, Nicolai Müller

**Affiliations:** 1 Department of Biology, University of Konstanz, Konstanz, Germany; 2 Graduate School Biological Sciences, University of Konstanz, Konstanz, Germany; 3 Department of Chemistry, University of Konstanz, Konstanz, Germany; University of Westminster - Regent Street Campus: University of Westminster, UNITED KINGDOM OF GREAT BRITAIN AND NORTHERN IRELAND

## Abstract

Anaerobic microbial degradation of the amino acids glycine and serine either occurs through enzymes of the so-called Stickland reaction or alternatively through the glycine cleavage system (GCS). In the mesophilic anaerobe *Peptoclostridium acidaminophilum*, initial glycine degradation proceeds through disproportionation to methylene tetrahydrofolate (THF) and acetyl-phosphate by GCS and glycine reductase. The thermophilic acetogen *Thermacetogenium phaeum* is able to utilize glycine, serine or threonine as sole carbon source, although it lacks genes for glycine reductase. In contrast, *T. phaeum* possesses genes for the GCS as well as for serine-converting enzymes, and the corresponding enzymes were specifically overabundant in the proteome and active. Among these enzymes, serine dehydratase was most active in serine-grown cells, even though its abundance in the proteome was comparably low. We suggest that two serine-converting enzyme systems (serine dehydratase, and the combination of glycine hydroxymethyltransferase and GCS) are used under different growth conditions: for breakdown of serine, *T. phaeum* most likely converts serine to pyruvate and ammonia by serine dehydratase, followed by acetate and ATP production via pyruvate dehydrogenase, phosphate acetyltransferase and acetate kinase. Electron carriers are then re-oxidized through CO_2_-fixation via the Wood-Ljungdahl pathway (WLP) of acetogenesis. When grown with glycine, the GCS most likely converts glycine to methylene-THF, which is then disproportionated to methenyl-THF and methyl-THF in the WLP. Glycine and serine both are excellent substrates for *T. phaeum*, yet in syntrophic cocultures with *Methanothermobacter thermautotrophicus*, acetate from glycine or serine degradation cannot be degraded further, as in syntrophic cultures with acetate as sole carbon source. This indicates an inhibitory or regulatory effect of glycine or serine degradation on acetate-degrading enzymes, resulting in the inability of *T. phaeum* to transition directly to syntrophic acetate oxidation after amino acid degradation.

## Introduction

The thermophilic acetogen *Thermacetogenium phaeum* is mainly known for its ability to grow with acetate as sole source of energy in syntrophic cocultures with *Methanothermobacter thermautotrophicus* [1]. The rare metabolic feature of anaerobic acetate oxidation classifies *T. phaeum*, besides *Clostridium ultunense*, *Thermotoga lettingae*, *Syntrophaceticus schinkii*, *Tepidanaerobacter acetatoxydans*, and ‘*Candidatus* Syntrophonatronum acetioxidans’ as one of the only few defined syntrophic acetate oxidizing bacteria (SAOB) described to date [1–6]. Moreover, *T. phaeum* degrades several C1 and C2 compounds under anaerobic conditions either in axenic cultures or in syntrophic cocultures with *M. thermautotrophicus* [1]. Recently, the metabolic pathways for C1 and C2 compounds (ethanol, ethanolamine, and methanol) were reconstructed by total proteome analysis and in vitro enzyme assays [7]. An additional study investigated the proteome change during syntrophic growth with acetate compared to axenic growth with hydrogen and CO_2_ or formate [8]. It was demonstrated that membrane-bound formate dehydrogenase, aldehyde oxidoreductase (AOR) and NAD^+^-independent methylene-tetrahydrofolate reductase (MTHFR) are active when *T. phaeum* grows with acetate in syntrophic coculture with *M. thermautotrophicus*. These enzymes in combination thus pose a system for saving ATP and for catalyzing a reversed electron transport to overcome the thermodynamic limitations of anaerobic breakdown of acetate [8]. However, this pathway may only be one of the several possible metabolic routes of syntrophic acetate oxidation, potentially varying amongst different SAOBs. Alternatively, the amino acid-converting serine dehydratase, serine (glycine) hydroxymethyltransferase, and the glycine cleavage system (GCS) were previously postulated to be potentially used in the mesophilic SAOB *Syntrophaceticus schinkii* for circumventing the endergonic reactions during syntrophic acetate oxidation [9,10]. Amino acids pose a less-known and less-investigated class of substrates for mesophilic and thermophilic acetogens, and only a limited number of examples have been reported. For example, the mesophilic model acetogen *Acetobacterium woodii* converts alanine to acetate and ammonia via pyruvate and acetyl-CoA and re-oxidizes electron carriers by CO_2_-reduction to acetate in the Wood-Ljungdahl pathway (WLP) [11]. On the other hand, glycine betaine is demethylated in *A. woodii*, transferring one methyl group to methyl-THF, which is then further disproportionated to acetate and CO_2_ by WLP enzymes [12]. Thermophilic amino acid degradation at 55°C was shown before in enrichment cultures involving four to five different morphological types of bacteria [13]. Moreover, thermophilic growth with glycine or cysteine as substrates was reported earlier for *T. phaeum* [1]. Yet, the biochemical pathways of glycine degradation are poorly investigated in both mesophilic and thermophilic acetogens. Even though genes of serine dehydratase, serine (glycine) hydroxymethyltransferase, and enzymes of GCS are present in the genome of *T. phaeum*, overabundance of these enzymes could not be verified in *T. phaeum* during syntrophic growth with acetate [8]. The apparent absence or underabundance of glycine- and serine-converting enzymes and glycine cleavage system in the acetate-grown proteome therefore prompted us to analyze the role of these enzymes in *T. phaeum*.

**Fig 1:**
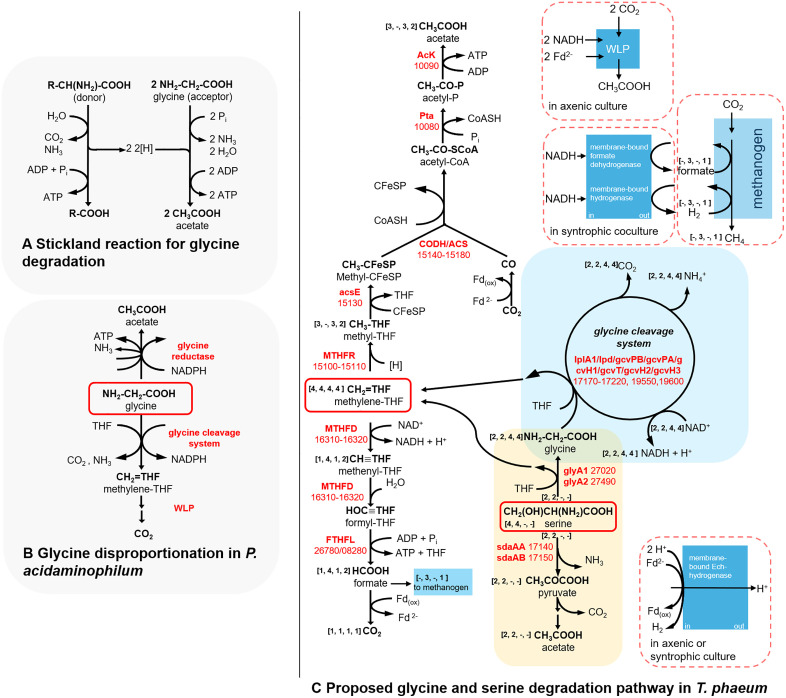
Amino acid degradation pathways. A: Mechanism of Stickland reaction for glycine degradation with another amino acid as electron donor [14]. B: Glycine disproportionation in *P. acidaminophilum* [15]. C: Proposed glycine and serine degradation pathway in *T. phaeum*. Numbers represent the IMG gene locus tags without prefix “Tph_c”. Abbreviations: WLP, Wood-Ljungdahl pathway; sdaAA, L-serine dehydratase; sdaAB, L-serine dehydratase; glyA1, glycine hydroxymethyltransferase; glyA2, serine hydroxymethyltransferase; lplA1, lipoate-protein ligase A; lpd, dihydrolipoamide dehydrogenase; gcvPB, glycine dehydrogenase (decarboxylating) beta subunit; gcvPA, glycine dehydrogenase (decarboxylating) alpha subunit; gcvH1, glycine cleavage system H protein; gcvT, aminomethyltransferase; gcvH2, glycine cleavage system H protein; gcvH3, glycine cleavage system H protein; THF, tetrahydrofolate; MTHFD, methylene-THF dehydrogenase; FTHFL, formyl-THF lyase; MTHFR, methylene-THF reductase; acsE, 5-methyltetrahydrofolate corrinoid/iron sulfur protein methyltransferase; AcK, acetate kinase; Pta, Phosphotransacetylase; CFeSP, corrinoidiron sulfur protein; CODH/ACS, carbon monoxide dehydrogenase/acetyl-coenzyme A synthase-complex; Fd_(ox)_, ferredoxin; Fd^2−^, reduced ferredoxin. Predicted stoichiometries of metabolites for the respective growth conditions are shown in square brackets in following order: [serine axenic, serine syntrophic, glycine axenic, glycine syntrophic]. In the modified WLP, methylene-THF is disproportionated to acetate and CO_2_. Reductive branch of WLP: upward-facing arrows, oxidative branch of WLP: downward-facing arrows.

In *Clostridium sticklandii*, glycine can act as an electron acceptor, where it is converted to acetate in the reductive branch of Stickland reactions involving other amino acids (Fig 1A, [14]). Additionally, it may be metabolized via GCS as observed in *Eubacterium acidaminophilum* [16]. In the mesophilic anaerobe *Eubacterium acidaminophilum*, which was reclassified as *Peptoclostridium acidaminophilum*, glycine can be disproportionated to acetate and methylene-THF by glycine reductase and GCS, respectively [15,17]. GCS catalyzes the conversion of glycine, NADP^+^, and tetrahydrofolate (THF) to methylene-THF, NH_3_, NADPH and CO_2_. Then, NADPH is re-oxidized by glycine reductase with another molecule of glycine while releasing NH_3_ and acetyl-phosphate, which is finally converted to acetate (Fig 1B) [15]. Methylene-THF, in turn, is converted to methenyl-THF, formyl-THF, formate, and finally CO_2_ through enzymes of the WLP of acetogenesis [15]. Excess reducing equivalents are then used to produce hydrogen or by reducing glycine to acetyl-phosphate and ammonia. Alternatively, when *P. acidaminophilum* grows in the presence of two substrates, i.e., glycine and other amino acids, excess reducing equivalents from glycine oxidation are likely transferred to the reduction of co-substrates, e.g., alanine [15]. When serine is the growth substrate for *P. acidaminophilum*, an L-serine hydroxymethyltransferase converts serine to glycine and methylene-THF, which are then further processed by the GCS and by enzymes of the WLP, respectively [15]. Hence, methylene-THF is the metabolite that links the reversible glycine cleavage system to the WLP either in the glycine-oxidizing direction or as a glycine synthase as described previously for *Clostridium drakei* [18].

In the current study, we demonstrate that *T. phaeum* grows with glycine, serine, or threonine as sole source of carbon and electrons either in pure (axenic) culture or in syntrophic coculture with *M. thermautotrophicus*. Furthermore, we have identified the key enzymes of amino acid degradation. Total proteome analysis and in vitro enzyme assays revealed that gene expression and activities of the corresponding enzymes are specifically induced during growth with glycine or serine as substrate. Modified biochemical pathways for the anaerobic, thermophilic degradation of glycine and serine, which slightly deviate from those of mesophilic systems, are postulated.

## Materials and methods

### Microorganisms and cultivation conditions

*T. phaeum* strain PB (DSM 26808) and *Methanothermobacter thermautotrophicus* strain TM (DSM 12269) were purchased from the German Culture Collection (DSMZ, Braunschweig, Germany). All cultures were incubated anoxically in modified freshwater medium, following the DSMZ protocol with medium 880 at 58 ± 5 °C and modifications as previously described [7,8]. Substrate stock solutions were sterilized by autoclaving for 30 min at 121 °C and 1 bar overpressure and added to the medium shortly before use. 40 mM acetate, 40 mM methanol, 40 mM ethanol, 20 mM ethanolamine, 10 mM glycine, 10 mM L-serine and 10 mM L-threonine were used for cultures of proteomics analysis. Depending on the purpose of the growth experiments, different concentrations of substrates were used. All of the abovementioned substrates were used in cultivating *T. phaeum* under axenic or syntrophic conditions, except for acetate, which was only used in syntrophic cultures. Approximately 70 mL of liquid cultures were maintained in 120 mL bottles sealed with butyl-stoppers, with headspace purged with N_2_/CO_2_ (80%/20%, v/v). Growth tests for assessing the inhibitory effect of ammonium were done in 25 mL glass tubes with butyl rubber stoppers sealed with aluminum crimps. To each tube, 100 µL double distilled water was added to establish steam pressure in the sealed tube. Then, the headspace was exchanged by repeatedly evacuating and gassing with N_2_/CO_2_ (80%/20%, v/v). The tubes were then autoclaved and, after cooling to room temperature, filled with 9.3 to 10 mL medium with 40 mM Na-acetate, depending on the volume of additions, to achieve a final volume of 10 mL. Varying concentrations of NH_4_Cl were added from a sterile, anoxic 1 M stock solution and the tubes were then inoculated with 500 µL of a preculture in stationary phase. Cultures were incubated at 61 °C and optical densities were recorded in regular intervals over time at 600 nm in a tube photometer M107 (Camspec, Leeds, United Kingdom) fitted with a tube holder to measure optical densities directly in culture tubes as described before [7].

### Cell lysis and subcellular fractionation

Cell lysis and subcellular fractionation were performed under strictly anoxic conditions as described earlier [7,8]. Anoxic cultures were transferred to suitable centrifuge bottles in an anoxic chamber filled with N_2_/H_2_ (95%/5%, v/v, Coy Laboratory Products, Ann Arbor, MI, United States). Cells were harvested at late exponential phase by centrifugation for 15 min at 7000 × g and 4 °C. The resulting pellets were washed three times with 50 mM potassium phosphate (KPP) buffer or Tris-HCl buffer (pH 7.5), containing 3 mM DTT. After the washing steps, the pellets were resuspended in 3–6 mL of buffer, depending on the size of pellets. The cells were disrupted by passaging through a French Press cell (Aminco, Silver Spring, MD, United States) at least three times and at a pressure of 137 MPa. The cell debris was separated from the soluble fraction by ultracentrifugation at an average, relative centrifugal field of r_av_ 93,900 × g (50,000 rpm), 4 °C for 60 min in an Optima^TM^ MAX-TL Ultracentrifuge using a TLA110-rotor (Beckman Coulter, Brea, CA, United States). Membrane and soluble fractions were kept on ice separately in sealed bottles purged with N_2_. Membrane fractions were prepared by redissolving the resulting pellets from the ultracentrifugation with 500 μL of the buffer used in assays. Samples were desalted and rebuffered by loading the soluble fraction onto a pre-equilibrated PD-10 desalting column packed with Sephadex™ G-25 resin (Cytiva) with the same buffer as in the washing steps. All manipulations were done according to the manufacturer’s instructions.

### Enzyme assays

All enzyme assays were performed under strictly anoxic conditions at 55 °C at least in triplicates. Controls with denatured protein fractions or abiotic controls without protein were performed in the same way as the experimental assays at least in triplicates. In discontinuous measurements for serine-converting enzymes (serine dehydratase and glycine hydroxymethyltransferase) and GCS enzymes, all reactants were combined in anoxic tubes with protein fractions in buffer (50 mM KPP, pH 7.5, containing 3 mM DTT). Samples withdrawn after 0, 30, 60, 120, or 180 minutes were prepared for amino acid or pyruvate measurements as described below. The change in reactants or products over time was used to determine enzyme activities. All the assays mentioned above were performed with protein fractions isolated from cultures grown with serine or acetate. Protein soluble fractions and desalted soluble fractions were prepared as described above. For continuous measurements of the GCS enzymes, MTHFR and NADH:acceptor oxidoreductase, assays were carried out in anoxic cuvettes flushed with N_2_/CO_2_ (80%/20%, v/v) in a Jasco V630 or V730 spectrophotometer fitted with thermostatted cuvette holders (Tokyo, Japan) heated to 55°C with a water bath. Assays were carried out in 50 mM Tris-HCl buffer (pH 7.5, containing 3 mM DTT), by continuously detecting the absorption of corresponding compounds.

### Serine-converting enzymes

The term “serine-converting enzymes” refers to serine dehydratase, glycine hydroxymethyltransferase and total serine-converting enzyme (sum of serine dehydratase and glycine hydroxymethyltransferase).

Serine-converting enzymes were assayed in one experiment mixture by determining the conversion of serine (for determination of the combined activity of total serine-converting enzymes), the production of pyruvate (for determination of serine dehydratase activity), and the production of glycine (for determination of glycine hydroxymethyltransferase activity). The reaction mixture was prepared by combining 20 mM serine and 0.5 mM THF with protein fractions in buffer. Samples were taken at 0, 30, 60, 120, and 180 minutes after the start of the reaction. Proteins were prepared separately from axenic cultures with 10 mM serine, 40 mM serine, syntrophic cultures with 10 mM serine and syntrophic cultures with 40 mM acetate. Desalted soluble fractions of each protein sample were used in these assays.

Serine dehydratase was assayed solely by mixing protein fractions with 20 mM serine in buffer. Pyruvate concentrations were measured by HPLC, and the production rate of pyruvate was used to calculate specific enzyme activities.

### Glycine cleavage system

The glycine cleavage system was assayed by both discontinuous measurement and continuous photometric measurement. In discontinuous enzyme assays, the soluble fraction obtained from cell-free extracts was mixed with 20 mM glycine, 0.5 mM THF and 2 mM NAD^+^ in buffer. Samples were withdrawn from the reaction mixture at 0, 30, 60, and 120 min after the reaction started. The concentration of glycine at each time point was measured to determine the conversion rate of glycine and, thus, the enzyme activities. In photometric enzyme assays, the glycine cleavage system was assayed with the same reactants in anoxic cuvettes. The reactants THF (0.5 mM) and glycine (20 mM) were pre-mixed with protein fractions. The reaction was started by adding NAD^+^ (1 mM). The reduction of NAD^+^ was followed at 340 nm [ε_340_ = 6.3 mM^−1^ cm^−1^ [19]].

### Methylene-THF reductase (MTHFR) and NADH:acceptor oxidoreductase

MTHFR and NADH:acceptor oxidoreductase were measured continuously with a spectrophotometer as described above. MTHFR was measured with 0.25 mM NADH as electron donor and 0.25 mM methylene-THF as electron acceptor. Methylene-THF was synthesized directly in the buffer by mixing 1.5 mM formaldehyde and 0.5 mM THF as described in [20]. Controls with formaldehyde alone were measured in the same way to compensate for a potential background reaction with formaldehyde. The oxidation of NADH was followed at 365 nm [ε_365_ = 3.441 mM^−1^ cm^−1^ [19]]. NADH:acceptor oxidoreductase was measured with 1 mM NADH and 0.25 mM anthraquinone-2,6-disulfonate (AQDS) as electron carrier. The reduction of AQDS was followed at 408 nm [ε_408_ = 7.2 mM^−1^ cm^−1^ [[21] Supporting Information]]. MTHFR and NADH:acceptor oxidoreductase were measured with soluble fraction and membrane fraction of cell-free extract of axenically grown cells with serine.

### TMT-labelling

Cells were collected at late exponential phase from various growth conditions: axenic or syntrophic cultures with ethanol, ethanolamine, glycine, serine or threonine as substrate, axenic cultures with methanol as substrate, and syntrophic cultures with acetate as substrate. Cells were opened with lysis buffer included in the TMTsixplex™ isobaric mass tagging kit (Thermo Fisher Scientific, Waltham, MA, USA) according to the manufacturer’s instructions. Protein concentrations were estimated using a Pierce™ BCA assay kit (Thermo Fisher Scientific, Waltham, MA, USA). Cell lysates were processed with a TMTsixplex™ isobaric mass tagging kit (Thermo Fisher Scientific, Waltham, MA, USA). Proteins in cell extracts were reduced, alkylated and digested overnight according to the manufacturer’s manual. Peptide concentrations were determined with a Pierce™ Quantitative Colorimetric Peptide Assay kit according to the instructions (Thermo Fisher Scientific, Waltham, MA, USA). Defined, normalized amounts of peptides of each sample were labelled with the six different mass tag reagents and then combined. Acetonitrile from labelling was removed by evaporating the combined, tagged samples with a vacuum centrifuge Univapo 100-H (Uniequip, München, Germany). Dried residues were resuspended in MilliQ-water and subjected to sample preparation and clean-up for MS analysis. Labelled samples were analyzed by LC-MS/MS before data analysis to identify peptides and quantify reporter ion relative abundance. Measurements of all conditions were performed in triplicate or quadruplicate.

### Mass spectrometric analysis of TMT-labelled samples

For sample preparation, all samples were reduced with DTT (30 min, 56 °C) and alkylated with chloroacetamide (60 min, RT). Digestions were performed using Trypsin (16 h, 30 °C). All digests were analysed on a QExactive HF mass spectrometer (Thermo Fisher Scientific, Bremen, Germany) interfaced with an Easy-nLC 1200 nanoflow liquid chromatography system (Thermo Fisher Scientific, Bremen, Germany). The peptide digests were reconstituted in 0.1% formic acid and loaded onto the analytical column (75 μm × 50 cm). Peptides were resolved at a flow rate of 150 nL/min using a gradient of 6 − 25% solvent B (0.1% formic acid in 80% acetonitrile) over 78 min, followed by increasing solvent B to 48% in another 30 min. Data-dependent acquisition with full scans in a 350 − 1500 m/z range was carried out at a mass resolution of 120000. The 15 most intense precursor ions were selected for fragmentation. Peptides with charge states 2 − 5 were selected, and dynamic exclusion was set to 30 sec. The fixed first mass was set to 100 m/z. Precursor ions were fragmented using higher-energy collision dissociation (HCD) set to 28%. For data evaluation, the raw data were evaluated using the MaxQuant software (version 2.4.2.0). Reporter MS2 with TMT 6plex was chosen as the type of LC-MS measurement. Other parameters were not changed. Identified proteins were listed in Microsoft Excel 2019 tables (Microsoft Corporation), along with the respective area values of corresponding peptides as a semi-quantitative measure of protein abundance as described before [7,8]. The raw data in the form of Excel tables can be found in the supplementary information (S1 - S3 Files). Statistical analysis and diagrams were prepared with R and MATLAB. Where indicated, -log_10_ p-values were calculated from two-sided t-tests and Volcano plots were generated using R version 4.2.3 and the ggplot2-package [22,23], with RStudio version 2025.05.0 + 496 [24]. Bar graphs were generated by MATLAB (version R2023a, MathWorks).

### Analytical methods

Acetate or pyruvate were quantified by HPLC with a Rezex^TM^ RHM-Monosaccharide H^+^ (8%) ion exchange resin column (LC column 300 x 7.8 mm, 00H-0132-K0, Phenomenex, Los Angeles, USA) operated at 40 °C and using an RID-20A refractive index detector (Shimadzu, Tokyo, Japan) essentially as described before [7]. Gas chromatography was used to quantify methane with a flame ionization detector (FID) and hydrogen with a thermal conductivity detector (TCD) in 500 µL culture headspace samples or calibration standards with an SGI 8610C (SRI Instruments, Los Angeles, USA) as previously described [7]. Glycine, serine, and threonine were derivatized and thereafter analyzed by HPLC as follows: cells in samples taken from cultures were separated from supernatant by centrifugation after mixing with acetonitrile in 1:1 volume ratio. Derivatization reagent was prepared freshly as described in previous studies [25]. Afterwards, the supernatant was mixed with derivatization reagent in 1:1 volume ratio and incubated at 4 °C for at least 30 min before loading onto an HPLC column. The eluent system consisted of eluent A (0.05 M sodium acetate, 10% acetonitrile, pH 7.2) and eluent B (0.1 M sodium acetate-acetonitrile-methanol in volume ratio of 46/44/10, pH 7.2) was adjusted as described without tetrahydrofuran [26]. Elution was performed in gradient mode as follows: initial conditions were 100% eluent A; from 8 to 16 minutes, the concentration of eluent B increased from 0 to 50%, and these conditions remained for 2 minutes. From 18 to 26 minutes, the conditions were changed back to 100% eluent A. Protein concentrations of cell lysates were estimated with a Pierce™ Protein BCA assay kit (Thermo Fisher Scientific, Waltham, MA, USA).

### Database analysis

Genomes of *Thermacetogenium phaeum* PB [27], *Peptoclostridium acidaminophilum* al-2 [28] and *Syntrophaceticus schinkii* Sp3 [29] were analyzed and genes compared using the IMG gene search tool and the IMG genome BLAST tool (https://img.jgi.doe.gov/cgi-bin/m/main.cgi) with the blastp program comparing amino acid sequences [30].

### Software

Besides the software mentioned in the “Mass spectrometric analysis of TMT-labelled samples” section and for routine data analysis such as preparation of growth curves, calculation of average values and standard deviations as well as for the preparation of the manuscript, Excel® 2019 and Word® 2019 as part of the Microsoft® Office 2019 package were used (Microsoft Corporation, https://www.microsoft.com).

### Sources of materials and chemicals

All chemicals and reagents were of analytical grade and used without further purification. Salts for medium preparation were purchased from Carl Roth (NaCl, KH_2_PO_4_, CaCl_2_·H_2_O, NH_4_Cl, Karlsruhe, Germany), or Merck (MgCl_2_ x 6 H_2_O, KHCO_3_, resazurin, Darmstadt, Germany). Substrates for cultivation were obtained from Carl Roth (acetate, glycine, serine), Merck (ethanolamine, threonine) or VWR (ethanol, methanol, Radnor, PA, USA). Compounds for the preparation of buffers and derivatization reagents were acquired from Carl Roth (KH_2_PO_4,_ H_2_SO_4_, sodium acetate), Merck (Phthaldialdehyde, K_2_HPO_4_ x 3 H_2_O, 3-mercaptopropionic acid), or VWR (acetonitrile). Gases had a purity of 99.999% and were obtained from Messer-Griesheim (Darmstadt, Germany) or Sauerstoffwerke Friedrichshafen (Friedrichshafen, Germany).

## Results

### Growth with amino acids as substrates

**Fig 2:**
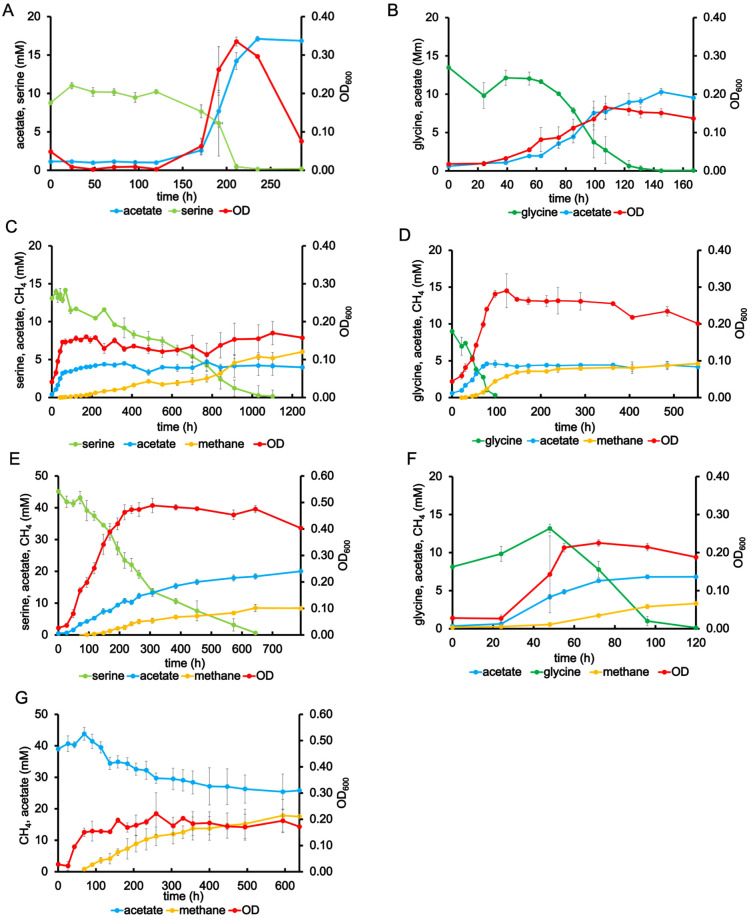
Growth of *T. phaeum* with glycine, serine or acetate as substrate. A: axenic culture with 10 mM serine at 61°C, B: axenic culture with 10 mM glycine at 61°C, C: syntrophic culture with 10 mM serine at 55°C, D: syntrophic culture with 10 mM glycine at 55°C, E: syntrophic culture with 40 mM serine at 55°C, F: syntrophic culture with 10 mM glycine at 61°C, G: syntrophic culture with 40 mM acetate at 61°C.

Cultures of *T. phaeum* grew exponentially or linearly under syntrophic or axenic conditions with glycine, serine, or threonine at 55°C or 61°C. Growth with threonine was much poorer, especially under axenic conditions. Acetate accumulated as a main product under all conditions, while methane was only detected in syntrophic cultures. H_2_ was not detected under any condition. Among all the cultures supplied with 10 mM substrates, the highest OD value (0.34) was observed in axenic cultures with serine at 61 °C (Fig 2A). In axenic cultures with glycine and serine, the doubling times were 35 h and 23 h, respectively (Table 1). Compared to growth with non-amino acid substrates, *T. phaeum* grew more slowly with serine or glycine than with methanol in axenic cultures (doubling time 12.5 h [7]) but in a similar range when compared to ethanol (doubling time 32 h [7]). Under syntrophic conditions, the doubling time of binary cultures with glycine at 55°C was 30 h, thus shorter than in syntrophic cultures with acetate as substrate (doubling time of binary cultures 42.4 h [7]).

**Table 1:** Stoichiometry and growth yields of amino acid degradation. Substrate degradation, growth yields and doubling times refer to total cultures or total biomass formation of either axenic *T. phaeum* or syntrophic, binary cultures of *T. phaeum* and *M. thermautotrophicus*. All growth experiments were carried out in triplicates. Cell dry weights were estimated using OD-values as a measure of total biomass formation of either *T. phaeum* alone or *T. phaeum* with *M. thermautotrophicus* with a theoretical correlation factor of 250 mg cell dry weight per 1L of a culture of OD_600_ = 1 as described before [7]. Assimilation equations: glycine: 17 C_2_H_5_NO_2_ + 14 H_2_O → 6 < C_4_H_7_O_3_ > + 10 H_2_CO_3_ + 17 NH_3_, serine: 17 C_3_H_7_NO_3_ + 12 H_2_O → 10 < C_4_H_7_O_3_ > + 11 H_2_CO_3_ + 17 NH_3_, threonine: 17 C_4_H_9_NO_3_ + 9 H_2_O → 16 < C_4_H_7_O_3_ > + 4 H_2_CO_3_ + 17 NH_3_. n.a. = not applicable, n.d. = not determined.

	Substrate degraded (mM)	Cell dry weight formed (mg/l)	Substrate assimilated (mM)	Products formed (mM)		Growth yield (g cell dry weight/mole substrate)	Electron recovery	Type of growth	Doubling time (h)
				acetate	methane				
**axenic culture**									
glycine 61 °C	13.46	37.5	1.03	10.12	n.d.	3.02	109%	exponential	35
serine 61 °C	10.85	72.5	1.20	16.12	n.d.	7.51	134%	exponential	23
**syntrophic culture**									
glycine 55 °C	8.99	62.5	1.72	4.89	3.05	8.60	146%	exponential	30
glycine 61 °C	13.05	50	1.38	6.47	3.14	4.28	110%	exponential	20
serine 55 °C (low concentration)	14.11	32.5	0.54	4.28	6.03	2.39	61%	linear	n.a.
serine 55 °C (high concentration)	45.17	115	1.90	19.54	8.7	2.66	52%	linear	n.a.

Although *T. phaeum* is a thermophilic bacterium with an optimum incubation temperature of 58°C [1], a temperature fluctuation of +/- 5 °C can influence the growth and substrate consumption. It was observed that *T. phaeum* had a higher growth rate at 61°C compared to 55°C, except for the axenic culture with glycine (S1A - S1F Figs, S16 Fig, S4 File). In syntrophic cultures, glycine was consumed within 120 hours at both temperatures (Figs 2D and 2F), while methane production was 0.42 and 0.27 mM per mM dissimilated glycine at 55°C and 61°C (methane production/dissimilated glycine was 3.05 mM/7.27 mM at 55°C and 3.14 mM/11.67 mM at 61°C, Table 1), respectively. This indicates that more acetate produced from glycine degradation was further converted to methane at 55°C than at 61°C (Figs 2D and 2F). This suggests that higher temperatures may not hinder amino acid utilization but rather inhibit the acetate-converting enzymes. In syntrophic acetate cultures, the OD_600_ at 55°C was twice as high as that at 61°C (S1E Fig). Moreover, 40 mM acetate was almost completely consumed within 20 days at 55 °C [7]. However, at 61 °C, only 37.5% of 40 mM acetate could be consumed within 26 days (Fig 2G). Even when incubated at 55°C, which is a more suitable temperature for acetate conversion, no significant change in the concentrations of acetate or methane was observed after glycine was consumed (Fig 2D), indicating that *T. phaeum* and its methanogenic partner could not directly shift their metabolism from amino acid degradation to acetate degradation in the same batch culture.

Under syntrophic conditions with 10 mM serine, OD_600_ increased immediately after transferring to fresh medium, reaching the stationary phase within 70 h, while only about 10% of the substrate were consumed (Fig 2C). Serine was mainly consumed during the stationary phase, without significant change in OD_600_ (Fig 2C). *T. phaeum* was incubated with 10 mM or 40 mM serine under syntrophic conditions to investigate whether higher serine concentrations enhance growth, thereby shifting the metabolism toward acetate conversion. OD_600_ and acetate concentration were approximately 3 times and 4.5 times higher in the cultures with 40 mM serine than with 10 mM serine (Figs 2C and 2E). However, methane production was 0.44 and 0.20 mM per mM serine (dissimilated) in cultures with 10 mM or 40 mM substrate, respectively. This indicates that a higher substrate concentration only results in more accumulated acetate, which is not further converted to methane (Figs 2C and 2E, Table 1).

Ammonium, a product of amino acid degradation, was expected to accumulate in the cultures. Syntrophic cultures with 40 mM acetate were only slightly affected by the addition of 5–20 mM NH_4_Cl. Cultures without additional NH_4_Cl or with 5 mM or 10 mM NH_4_Cl, grew faster and reached the stationary phase between 50 and 75 hours, with final average optical densities of 0.27 to 0.28. Cultures with 20 mM NH_4_Cl reached the stationary phase after roughly 100 hours, with a somewhat lower final average optical density of 0.25 (S2 Fig).

### Serine-converting enzymes in *T. phaeum*

**Fig 3:**
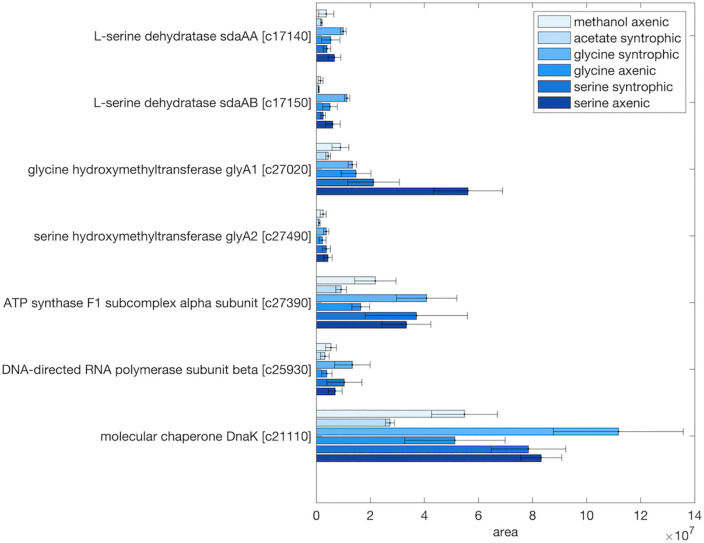
Proteome data of serine dehydratase and glycine hydroxymethyltransferase under various growth conditions of *T. phaeum.* Samples from different growth conditions were labelled with different TMT-labels and then mixed for further preparation and analysis. Shown are non-normalized area values compared to the area value of housekeeping proteins (ATPase, RNA-polymerase and molecular chaperone DnaK). Locus tags are shown without “Tph_” prefix (e.g., Tph_c17140 is shown as c17140). Shown are mean values from triplicate measurements + /- standard deviations.

The genome of *T. phaeum* harbors two possible serine-converting enzyme systems, i.e., serine dehydratase and glycine hydroxymethyltransferase. Both were found in the proteome under all growth conditions in varying abundance (S1 - S3 Files). Serine dehydratase had a relatively low area value compared to housekeeping genes under all growth conditions, indicating that serine dehydratase is not abundant in terms of protein amount (Fig 3). On the contrary, glycine hydroxymethyltransferase (glyA1) was more abundant, but mainly during growth with amino acids as substrates, indicating that glycine hydroxymethyltransferase might play a more important role in amino acid degradation in *T. phaeum* (Fig 3).

To assess the potential turnover rates of the two enzyme systems, serine dehydratase, glycine hydroxymethyltransferase and total serine-converting enzymes were assayed in one reaction mixture by determining the change in pyruvate, glycine and serine concentrations over time (S3A and S3B Figs). About 20 mM serine was consumed within 180 min in the assay for serine axenic culture, resulting in the production of 19.5 mM pyruvate and 1.31 mM glycine (S3A and S3B Figs). In the assay for serine syntrophic culture, only about 4 mM serine was consumed, producing 5.3 mM pyruvate and 0.5 mM glycine (S3B Fig). In the assay for acetate syntrophic culture, almost no serine degradation could be observed (S3A Fig). After subtracting the background reaction activities, the specific enzyme activities of serine dehydratase and glycine hydroxymethyltransferase were, on average, 0.55 and 0.14 U/mg desalted soluble protein fraction of axenic culture with 10 mM serine. The same assays were repeated with protein prepared from axenic culture with 40 mM serine. The resulting enzyme activities of the aforementioned enzymes were at a similar level, 0.56 and 0.03 U/mg, respectively (Table 2). Serine dehydratase was assayed individually for serine axenic culture and acetate syntrophic culture in the reaction mixture of desalted protein fraction and serine as the only reactant. Pyruvate production was only observed in the assay for serine axenic culture, with an average specific enzyme activity of 0.39 U/mg (Table 2, S15 Fig).

**Table 2:** Enzyme activities of *T. phaeum* grown with different substrates. The measured parameter in each assay system is shown in bold.

Culture conditions	Activity [U/mg]	Measured parameter	Substrates and corresponding reactions
glycine cleavage system			
40 mM serine axenic	0.23 ± 0.08	glycine consumption	20 mM glycine, 0.5 mM THF, 2 mM NAD^+^
40 mM acetate syntrophic	0.06 ± 0.02		**glycine** + NAD^+^ + THF → methylene-THF + CO_2_ + NH_3_ + NADH + H^+^
serine dehydratase			
40 mM serine axenic	0.39 ± 0.02	pyruvate production	20 mM serine
40 mM acetate syntrophic	-0.02 ± 0.03		serine → **pyruvate** + NH_4_^+^
serine-converting enzymes:			
serine dehydratase			
40 mM serine axenic	0.56 ± 0.03	pyruvate production	20 mM serine, 0.5 mM THF
40 mM acetate syntrophic	-0.04 ± 0.03		
10 mM serine axenic	0.55 ± 0.06		serine dehydratase:
10 mM serine syntrophic	0.12 ± 0.02		serine → **pyruvate** + NH_4_^+^
glycine hydroxymethyltransferase			
40 mM serine axenic	0.03 ± 0	glycine production	glycine hydroxymethyltransferase:
40 mM acetate syntrophic	no glycine production		serine + THF → **glycine** + methylene-THF
10 mM serine axenic	0.14 ± 0.05		
10 mM serine syntrophic	0.03 ± 0		total serine-converting enzymes:
total serine-converting enzymes			**serine** → pyruvate + NH_4_^+^
40 mM serine axenic	0.64 ± 0.014	serine consumption	**serine** + THF → glycine + methylene-THF
40 mM acetate syntrophic	-0.14 ± 0.13		
10 mM serine axenic	0.49 ± 0.11		pyruvate, glycine and serine were measured in samples taken from one reaction mixture
10 mM serine syntrophic	0.31 ± 0.14		

### The role of the glycine cleavage system in the metabolism of *T. phaeum*

**Fig 4:**
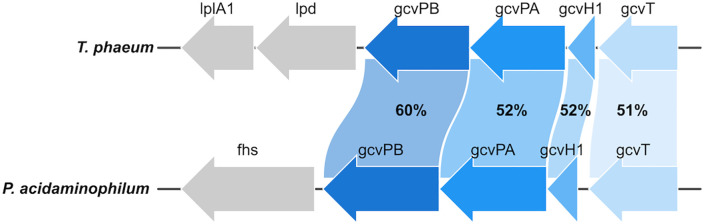
Comparison of the GCS gene clusters in *T. phaeum* and *P. acidaminophilum.* The numbers show the amino acid identities of the corresponding genes. The graph was drawn using ChiPlot (https://www.chiplot.online/).

The glycine cleavage system (GCS) (EC 1.4.1.27) consists of four components, i.e., a glycine decarboxylase gcvP (P-protein), an aminomethyltransferase gcvT (T-protein), a dihydrolipoamide dehydrogenase gcvL (L-protein), and a lipoic acid-containing carrier protein gcvH (H-protein) [31]. A complete set of genes for a GCS is present in the genome of *T. phaeum* in a putative operon, i.e. gcvT (IMG-locus tag Tph_c17220), gcvH1 (Tph_c17210), the two alpha and beta subunits of gcvP (gcvPA: Tph_c17200, gcvPB: Tph_c17190), as well as gcvL (lpd: Tph_c17180) and an associated lipoate-protein ligase (lplA1: Tph_c17170). Moreover, two genes annotated as glycine cleavage system H protein (gcvH2, Tph_c19550 and gcvH3, Tph_c19600) are located in a separate operon with lipoate protein ligase genes (Tph_c19540 and Tph_c19560) and genes annotated as pyruvate dehydrogenase E1 component alpha subunit (Tph_c19650), pyruvate dehydrogenase E1 component beta subunit (Tph_c19640), and pyruvate dehydrogenase E2 component (dihydrolipoyllysine-residue acetyltransferase) (Tph_c19620). An IMG Gene neighborhood search revealed that the most closely related gene cluster is the one in *Syntrophaceticus schinkii*. Each individual gene for the GCS in the same operon of *T. phaeum* was analyzed in an IMG Genome BLAST against the genome of *Peptoclostridium acidaminophilum*. These genes appear only distantly related to the corresponding genes of the Clostridial GCS in *P. acidaminophilum*, with amino acid identities for gcvT (IMG-locus tag EAL2_c17210), gcvH (EAL2_c17200), gcvPA (EAL2_c17190), and gcvPB (EAL2_c17180) between 51% and 60% (Fig 4). Homologues for gcvL (lpd) and lplA1 were not identified in the corresponding gene region of the genome of *P. acidaminophilum*. Instead, a gene for formate-tetrahydrofolate ligase fhs (EAL2_c17170) is located next to the genes for gcvP and genes somewhat related to gcvL (between 27% and 35% identity) or lplA1 (26% to 39% identity) can be found at different loci in the genome. However, none of the annotations of the genes identified for gcvL in *P. acidaminophilum* (mercuric reductase EAL2_c06150, NADPH-dependent 2,4-dienoyl-CoA reductase sulfur reductase EAL2_808p03500 and EAL2_c16730, CoA-disulfide reductase EAL2_808p03290, Pyridine nucleotide-disulphide oxidoreductase EAL2_c18070) hint towards a dihydrolipoamide dehydrogenase.

**Fig 5:**
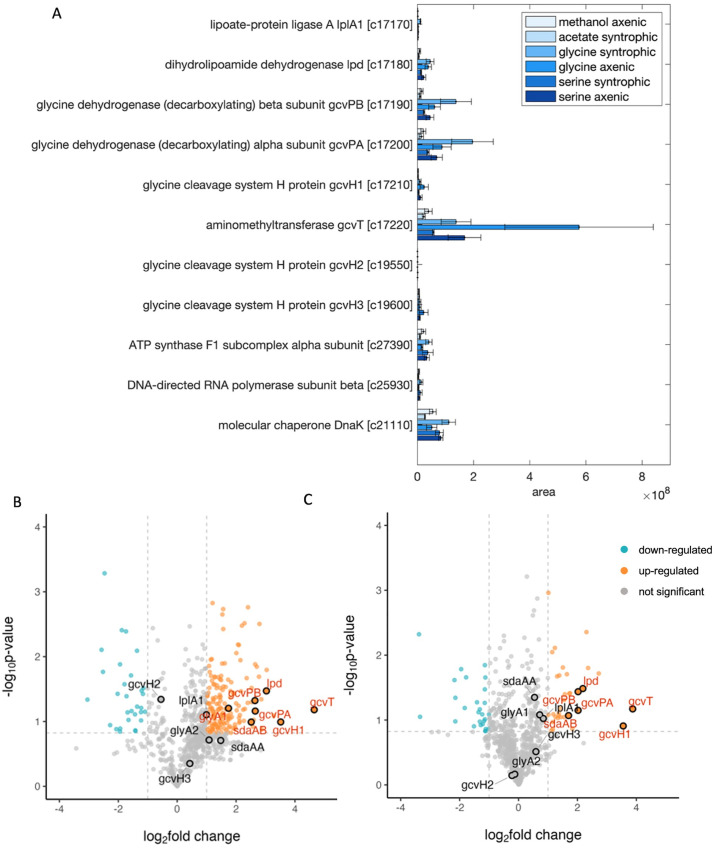
(A) Proteome data of the GCS under various growth conditions of *T. phaeum*. Samples from different growth conditions were labelled with different TMT-labels and then mixed for further preparation and analysis. Shown are non-normalized area values compared to the area value of housekeeping proteins (ATPase, RNA-polymerase and molecular chaperone DnaK). Locus tags are shown without “Tph_” prefix (e.g., Tph_c17170 is shown as c17170). (B) and (C) are volcano plots of glycine axenic conditions compared to acetate syntrophic conditions (B) and methanol axenic conditions (C). Serine dehydratase (sdaAA, sdaAB), glycine hydroxymethyltransferase (glyA1, glyA2) and GCS genes (lplA1, lpd, gcvPB, gcvPA, gcvH1, gcvT, gcvH2, gcvH3) are highlighted in black circles. Log2fold change > 1 or <−1, p-value<0.15 were selected as up- or down-regulated genes. Data were obtained from triplicate measurements.

Total proteome analysis revealed that the corresponding proteins were mainly produced during growth with glycine or serine (Fig 5). GcvT was significantly overabundant during axenic growth with glycine, with the area of 5.8 × 10^8^, which had the highest fold change compared to the growth with non-amino acid substrates (Figs 5A, 5B and 5C). Lpd, gcvPA and gcvPB were more abundant, mainly in the growth with amino acid as substrates (Fig 5A). LplA1, gcvH1, gcvH2 and gcvH3 were not abundant under all the conditions (Fig 5A). Although all components of the GCS were present in the genome of *T. phaeum*, not all of them were highly expressed during growth with amino acids.

Activities of the GCS were assayed by combining soluble protein fraction with glycine, THF and NAD^+^. By continuous photometric measurements, the absorption at 365 nm was measured, and, thus, the change of NADH was followed. However, the change in NADH concentration was not easily observed in this assay (S1 Table). The background absorption of the reaction mixture at 365 nm or 340 nm was too high due to the relatively high absorption of THF at these wavelengths. Moreover, the presence of MTHFR could possibly have caused the oxidation of NADH back to NAD^+^ with methylene-THF produced by the GCS making it difficult to assess the activity of the GCS using this method. With this method, a specific enzyme activity of 0.001 ± 0.00092 U/mg was observed in the soluble fraction of the serine axenic culture (S1 Table). To improve the enzyme assay of GCS, the glycine degradation rate was used to determine the activity. Only a slight decrease in glycine could be observed in the soluble fraction of the cell-free extract isolated from an axenically grown culture with serine: 17 mM glycine continuously decreased to 12 mM within 120 min (S4 Fig). After subtracting the background reaction, this corresponds to 0.23 U/mg (Table 2). However, the results in controls with denatured protein fraction and abiotic controls developed in a non-linear way, which was possibly caused by non-enzymatic reactions, i.e., release of glycine from denatured protein. Similar problems also occurred in the assay with protein soluble fraction isolated from syntrophic acetate culture. No continuous decrease of glycine was observed in these reaction mixtures. In these cases, only the start and end concentrations of glycine were used to determine enzyme activities, resulting in a specific enzyme activity of 0.06 U/mg in the assay for acetate syntrophic culture (Table 2).

### Hypothetical acetate oxidation pathway via the glycine cleavage system

Three prerequisites are needed for an acetate oxidation pathway involving the GCS as proposed earlier. These are serine dehydratase (EC 4.3.1.17) and glycine hydroxymethyltransferase (EC 2.1.2.1) for the conversion of acetate-derived pyruvate into glycine [9]. Then, the GCS (EC 1.4.1.27) converts glycine, THF, and NAD^+^ into methylene-THF, CO_2_, and NADH [9]. The genome of *T. phaeum* contains two genes annotated as serine dehydratase alpha-subunit and beta-subunit (sdaAA and sdaAB, IMG-locus tags Tph_c17140 and Tph_c17150), as well as two genes for L-serine hydroxymethyltransferase (EC 2.1.2.1, a.k.a. glycine hydroxymethyltransferase) (glyA1 and glyA2, IMG-locus tags Tph_c27020 and Tph_c27490).

The abovementioned glyA1 and glyA2 genes were present in the proteome of *T. phaeum* during growth with non-amino acid substrates, such as acetate, ethanol, ethanolamine and methanol, with low expression levels (S5 Fig). Comparison of the proteomes of amino acid-grown cells to non-amino acid-grown cells revealed that two genes of serine dehydratase (sdaAA and sdaAB) and one of the glycine hydroxymethyltransferase (glyA2) were not abundant in the proteome under any of these growth conditions (Fig 3). The other glycine hydroxymethyltransferase (glyA1) was more abundant when grown with serine under axenic conditions, with an average area of 5.6 × 10^7^, which was about 3 times higher than the average of other conditions and at a comparable level with housekeeping genes. Yet, this gene was moderately abundant with serine under syntrophic conditions (2.1 × 10^7^) or with glycine (1.5 × 10^7^ and 1.3 × 10^7^ under syntrophic and axenic conditions, respectively). However, this gene was not abundant when grown with non-amino acid substrates (methanol or acetate) (Fig 3). As judged by proteome analysis, it appeared that glycine hydroxymethyltransferase (glyA1) is primarily used for amino acid degradation. In contrast to the in vitro enzyme assays, glyA1 was more abundant in amino acid degradation than sdaAA and sdaAB, according to proteome analysis (Fig 3 and Table 2).

### Glycine reductase

One of the key enzymes involved in glycine or serine degradation in *P. acidaminophilum* is glycine reductase (EC 1.21.4.2), which was previously shown by enzyme assays and genome analysis [15,28]. A keyword search for the terms “glycine” or “glycine reductase” using the gene search tool of the IMG-database resulted in 15 hits for genes annotated as “glycine reductase”, “Glycine reductase complex selenoprotein A”, or “grdX; glycine reductase complex protein GrdX” in the publicly available genome sequence of *P. acidaminophilum* (EAL2_c02900, EAL2_c02910, EAL2_c08730, EAL2_c08740, EAL2_c08750, EAL2_c16970, EAL2_c17000, EAL2_c17010, EAL2_c17020, EAL2_c17030, EAL2_808p05070, EAL2_808p05080, EAL2_808p07350, EAL2_808p07380, EAL2_808p07390). The latter 15 genes constitute subunits of the glycine reductase, betaine reductase or sarcosine reductase. No hits were obtained after a search for the same keywords in the genome sequence of *Thermacetogenium phaeum* [27,28]. Database searches with the NCBI BLASTP tool using the amino acid sequences of the abovementioned 15 glycine reductase genes from *P. acidaminophilum* was performed against the genome of *T. phaeum*, yielding no significant hits. Based on these database analyses, we concluded that glycine reductase is not encoded in the genome sequence of *T. phaeum*.

### Proteome during growth with threonine

In the proteome of *T. phaeum*, two genes of threonine synthase (Tph_c27010 and Tph_c15550) were found to be moderately abundant during growth with threonine (S7 Fig). One of these threonine synthases (Tph_c27010) is located on a putative gene cluster adjacent to the gene of glycine hydroxymethyltransferase (glyA1, Tph_c27020). Remarkably, during syntrophic growth with threonine, a ketol-acid reductoisomerase (Tph_c19850) had the highest fold change compared to syntrophic growth with serine, and also among the genes with top fold change compared to other conditions (S8 Fig). Compared to the proteome in other growth conditions, the membrane-bound formate dehydrogenase genes (Tph_c15380–Tph_c15410) were upregulated in syntrophic growth, including in the syntrophic growth with threonine (S9 - S11 Figs). Moreover, in the same gene cluster of the membrane-bound formate dehydrogenase, two genes annotated as 4-hydroxy-3-polyprenylbenzoate decarboxylase (Tph_c15440 and Tph_c15450) were most highly abundant during syntrophic growth with threonine compared to other growth conditions (S9 Fig).

### Wood-Ljungdahl pathway enzymes

**Fig 6:**
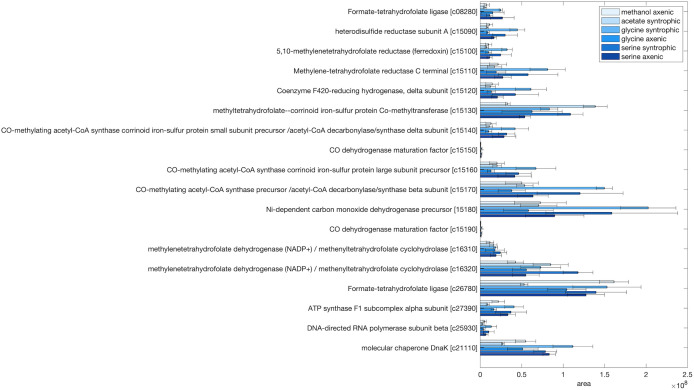
Proteome data of Wood-Ljungdahl pathway enzymes under various growth conditions of *T. phaeum.* Samples from different growth conditions were labelled with different TMT-labels and then mixed for further preparation and analysis. Shown are non-normalized area values compared to the area value of housekeeping proteins (ATPase, RNA-polymerase and molecular chaperone DnaK). Locus tags are shown without “Tph_” prefix (e.g., Tph_c08280 is shown as c0828. Shown are mean values from triplicate measurements + /- standard deviations.

Methylene-THF is one of the intermediates in Wood-Ljungdahl pathway (WLP). In the glycine disproportionation pathway of *P. acidaminophilum*, methylene-THF was one of the products of the GCS and further converted to CO_2_ through WLP enzymes [15]. The genome of *T. phaeum* contains all genes for a WLP [27]. Total proteomics analysis showed that all WLP enzymes were present under all growth conditions in this work. Except for the genes for CO dehydrogenase maturation cofactor (Tph_c15150 and Tph_c15190), which had area values of approximately 0, all other genes of WLP enzymes showed high abundance in most growing conditions (Fig 6). The key enzymes, such as the bifunctional methylenetetrahydrofolate dehydrogenase (NADP^+^)/methenyltetrahydrofolate cyclohydrolase (Tph_c16310 and Tph_c16320), which catalyze the conversion of methylene-THF to formyl-THF via methenyl-THF, acetyl-CoA synthase and carbon monoxide dehydrogenase (Tph-c15140, Tph_c15160 and Tph_c15170) were found abundant during growth with amino acids and other non-amino acid substrates (Fig 6 and S12 Fig). One subunit of the key enzymes, methylene-THF reductase (MTHFR, Tph_c15110), was found to be more abundant than the other subunit Tph_c15100, under all growth conditions (Fig 6).

MTHFR and NADH:acceptor oxidoreductase were assayed in soluble and membrane fractions of cell-free extracts isolated from axenically grown cells with 40 mM serine as substrate. MTHFR was assayed in the direction of methyl-THF formation with methylene-THF as educt. The metabolic pathway we propose in the current study suggests that methylene is disproportionated by MTHFR and MTHFD to form methyl-THF and methenyl-THF, respectively, with electrons transferred from MTHFD to MTHFR. However, electrons derived from NADH-oxidation in the form of a hydride (H^-^) would have to be transferred to an electron-only carrier to allow the NAD^+^-independent reduction of methylene-THF to methyl-THF observed before [7]. In assays with NADH as electron carrier, NADH oxidation with methylene-THF by MTHFR was mainly detected in the soluble fraction of cell-free extract with a corresponding low specific enzyme activity of 0.0222 U/mg (Table 3). In the MTHFR assays for syntrophically grown cells with acetate, enzyme activity was only detected in the subcellular fraction separated by anion exchange chromatography with a HiTrapQ column and eluted with 1 M NaCl [8]. NADH:acceptor oxidoreductase was assayed with AQDS as electron carrier. In syntrophically grown cells with acetate, the activity of NADH:AQDS oxidoreductase was detected exclusively in the soluble fraction [8]. In axenically grown cells with serine, AQDS reduction was detected in both soluble and membrane fractions, with corresponding specific enzyme activity of 0.0137 and 0.0788 U/mg, respectively (Table 3), meaning that the activity is mainly in the membrane fraction under this growth condition.

**Table 3:** Specific enzyme activities of methylene-THF reductase and NADH:acceptor oxidoreductase in axenically grown cells with serine.

	Electron carrier	Soluble fraction (U/mg)	Membrane fraction (U/mg)
methylene-THF reductase	NADH	0.0222 ± 0.0153	0.0003 ± 0.00322
NADH:acceptor oxidoreductase	AQDS	0.0137 ± 0.0081	0.0788 ± 0.0129

## Discussion

Amino acid metabolism in *T. phaeum* likely involves the GCS and WLP connected by methylene-THF. In this study, *T. phaeum* was able to grow in both axenic and syntrophic cultures with serine, glycine, or threonine as substrates, exhibiting an exponential or linear growth pattern, depending on the substrate and growth temperature. Growth in axenic cultures with glycine, serine, or threonine proceeded roughly according to the stoichiometries outlined in equations 1–3.


$$\begin{array}{c} \text{glycine},\text{axenic}:\text{4}C_{\text{2}}H_{\text{5}}{NO}_{\text{2}}+\text{2}H_{\text{2}}O\to \text{3}C_{\text{2}}H_{\text{3}}O_{\text{2}}^{-}+\text{3}H^{+}\;+\text{4}{NH}_{\text{3}}+\text{2}{CO}_{\text{2}} \end{array}$$ (1)

$$\begin{array}{c} \text{serine},\text{axenic}:\text{4}C_{\text{3}}H_{\text{7}}{NO}_{\text{3}}+\text{2}H_{\text{2}}O\to \text{5}C_{\text{2}}H_{\text{3}}O_{\text{2}}^{-}+\text{5}H^{+}\;+\text{4}{NH}_{\text{3}}\;+\text{2}{CO}_{\text{2}} \end{array}$$ (2)

$$\begin{array}{c} \text{threonine},\text{axenic}:C_{\text{4}}H_{\text{9}}{NO}_{\text{3}}+H_{\text{2}}O\to \text{2}C_{\text{2}}H_{\text{3}}O_{\text{2}}^{-}+\text{2}H^{+}\;+{NH}_{\text{3}} \end{array}$$ (3)

Besides the routine incubation of *T. phaeum* at 55°C, 61°C was also studied, originally due to an incidental observation of uneven incubator heating, under which acetate consumption and methane production were remarkably reduced compared to previous results. In syntrophic cultures, we found that higher temperatures may not significantly affect glycine or serine consumption, but likely affect the activity of the acetate utilization enzymes (S1 Fig). Even though it was incubated under the optimum temperature, *T. phaeum* could not completely mineralize glycine or serine to CO_2_. We found that, regardless of temperature and substrate concentration, the ratio of consumed glycine or serine to acetate produced in most cases was nearly 2:1. We therefore postulate theoretical stoichiometries of glycine and serine degradation in syntrophic culture as in equations 4 and 5.


$$\begin{array}{c} \text{glycine},\text{syntrophic}:\text{4}C_{\text{2}}H_{\text{5}}{NO}_{\text{2}}+\text{2}H_{\text{2}}O\to \text{2}C_{\text{2}}H_{\text{3}}O_{\text{2}}^{-}+\text{2}H^{+}\;+{CH}_{\text{4}}+\text{4}{NH}_{\text{3}}+\text{3}{CO}_{\text{2}} \end{array}$$ (4)

$$\begin{array}{c} \text{serine},\text{syntrophic}:\text{4}C_{\text{3}}H_{\text{7}}{NO}_{\text{3}}+\text{2}H_{\text{2}}O\to \text{2}C_{\text{2}}H_{\text{3}}O_{\text{2}}^{-}+\text{2}H^{+}\;+\text{3}{CH}_{\text{4}}+\text{4}{NH}_{\text{3}}+\text{5}{CO}_{\text{2}} \end{array}$$ (5)

In some cases, e.g., at a substrate concentration of 40 mM serine, the concentration of methane in our growth experiments was lower compared to the theoretical equations, and the electron recoveries were incomplete, hinting at unknown side reactions or growth inhibition. Apparently, these cultures did not thrive well under amino acid-grown conditions, which might also be the reason why syntrophic cultures did not immediately transition to acetate utilization once glycine or serine were completely consumed. Previous studies have shown that ammonia released from amino acid degradation may inhibit methanogens as well as SAOB, depending on the species and the concentrations of ammonia [32]. However, SAOB are supposed to be more susceptible to high ammonia compared to methanogens. The mesophilic SAOB *Tepidanaerobacter acetatoxydans* tolerates ammonium concentrations of 5 g/l, while *T. phaeum* is somewhat affected at 0.26 g/l and strongly inhibited already at 3 g/l over an observation period of 160 days [32]. Other authors have determined the maximum tolerable ammonium concentration to 1.0 M NH_4_Cl (18 g/l for ammonium) for *T. acetatoxydans* [5]. In this study, ammonium concentrations in the culture were estimated based on two sources: ammonium from amino acid degradation and ammonium in the basal medium, according to DSMZ medium protocol 880 (1 g/L ammonium chloride, corresponding to 19 mM or 0.34 g/L ammonium). Assuming a 1:1 ratio of amino acid to ammonia (40 mM serine to 40 mM or 0.72 g/l ammonium), ammonia/ammonium concentrations in the cultures in this study were presumably not higher than 60 mM (1.06 g/l ammonium). In syntrophic cultures with 10 mM serine, ammonium concentrations were supposedly lower, assuming 0.34 g/l ammonium from the basal medium plus 0.18 g/l ammonium maximally released from 10 mM serine (0.52 g/l). Our own growth inhibition studies with 40 mM acetate and 20 mM NH_4_Cl (0.36 g/l ammonium) showed only a slight inhibition of syntrophic acetate oxidation. The underlying reason why *T. phaeum* failed to transition to the energetically unfavorable acetate oxidation after the depletion of glycine or serine in syntrophic culture remains unclear. It is conceivable that other secondary products, such as primary amines, were released during amino acid degradation, which were inhibitory for the SAOB or the methanogen. Such secondary products could possibly occur from the activity of glycine- or serine-deaminating enzymes. These enzymes of the central metabolic pathways were further investigated.

All genes and the corresponding enzymes for known pathways for the breakdown of glycine or serine were identified in the genome and proteome of *T. phaeum*. However, glycine reductase, which was reported as a key enzyme in amino acid degradation in *P. acidaminophilum*, was absent in *T. phaeum*. One reason could be a direct consequence of growth temperature and the narrow temperature range of *T. phaeum*, as glycine reductase is a relatively temperature-sensitive protein. Earlier studies have shown that the activity of the so-called protein C, which is one of the three subunits A, B, and C of the clostridial glycine reductase, lost 30% of its activity after heating to 47°C for 10 min, while 80% of activity was lost after heating to 68°C for 10 min [33,34]. In addition, the product of glycine reductase, acetyl-phosphate, is somewhat unstable at elevated temperatures. In vitro studies have quantified the abiotic degradation of acetyl-phosphate, where 300 mM of this metabolic intermediate was completely hydrolyzed within 90 min at 60°C, while only 20% of the same amount of acetyl-phosphate was degraded within 5 hours at 20°C [35]. Yet, this thermal instability might possibly be overcome by a high-affinity turnover of acetyl-phosphate, depending on the enzymes further downstream of the pathway. It was shown, for instance, that phosphate acetyltransferase, and consequently its metabolite acetyl-phosphate, are involved in the metabolism of *T. phaeum* during growth with various substrates [7,8]. Although the temperature sensitivity of clostridial glycine reductase has been tested and confirmed, genes for glycine reductase were identified in thermophiles as well, such as *Clostridium tepidiprofundi* [36]. However, *C. tepidiprofundi*, a bacterium from deep-sea hydrothermal vents, apparently does not utilize glycine [37]. Glycine reductase may be only relevant at lower temperatures, as glycine is not utilized by *C. tepidiprofundi* at the optimum temperature of 50°C [37]. Compared to the broad temperature range (20–60°C) of *C. tepidiprofundi*, *T. phaeum,* in contrast, has a narrower temperature range of 40°C to 65°C and might therefore not be able to produce a functional glycine reductase, even if the respective genes were present in the genome [1,27]. The fact that *T. phaeum* does not possess genes for glycine reductase alone does, therefore, not necessarily indicate a temperature restriction, and the reason for the absence of this enzyme system in *T. phaeum* remains enigmatic.

Environmental stressors such as heat, salinity, aridity and acidity have shaped the genomes of environmental bacteria, such as the soil bacterium *Bradyrhizobium diazoefficiens* [38]. For instance, it was demonstrated that genome size negatively correlates with growth temperature as a reduced genome size lowers the volume of a cell, which favors survival at elevated temperatures [39]. The genome size of the thermophilic acetogen *T. phaeum* (2.94 Mbp [27]) is rather small in comparison to the mesophilic model acetogen *Acetobacterium woodii* (4.04 Mbp [40]). In *A. woodii*, Rnf-complex is considered an important enzyme system for energy conservation. Rnf catalyzes the oxidation of NADH with ferredoxin while simultaneously consuming a sodium ion gradient across the cytoplasmic membrane, thus posing an energy-converting enzyme system that allows the cell to drive endergonic redox reactions [41]. In contrast, the genome of *T. phaeum* lacks the genes for the six subunits of the Rnf-complex and uses smaller energy-converting enzyme systems [7]. It is therefore likely that, as a result of genome size reduction, *T. phaeum* harbors metabolic pathways that deviate from mesophilic models and that the thermophilic metabolism has to be more efficient in terms of proteins necessary per metabolic pathway. We have previously postulated such alternative metabolic pathways for *T. phaeum* [7]. Analogously, we present in the current study another alternative, thermophilic metabolic pathway for the anaerobic degradation of glycine and serine in the absence of glycine reductase (Fig 1C). The suggested pathway is similar to the model organism but also uses enzymes that differ from those in the glycine degradation pathway in the mesophilic *P. acidaminophilum*. The glycine degradation pathway of *P. acidaminophilum* suggests a disproportionation of glycine to methylene-THF and acetate by the glycine cleavage system and glycine reductase, respectively [15]. Differently, glycine reductase is missing in *T. phaeum*, and the disproportionation reaction is located further downstream in the pathway, at the level of methylene-THF, catalyzed by two WLP enzymes: methylene-THF reductase and methylene-THF dehydrogenase (Fig 1C). Hence, it appears that glycine degradation proceeds by a slightly altered pathway in *T. phaeum*, which might, among other reasons, be due to a growth temperature-induced, reduced genome size.

Apparently, GCS enzymes play a central role in amino acid metabolism in *T. phaeum*, as they are more abundant in the proteomes of cultures with amino acid as substrate than in cultures with non-amino acid substrates. Although all four components of the GCS were found in the proteome of *T. phaeum* grown with amino acids, one of these four components, the lipoic acid-containing carrier protein gcvH (H-protein), was not abundant in all cases, with areas lower than under all growth conditions (Fig 5). H protein acts as a connector of the loosely bound complex of P-protein, T-protein and L-protein, undergoing various changes in the forms among oxidized, reduced and aminomethylated forms by interacting with other three components of GCS. A kinetic study of H-protein showed that H-protein has a significantly higher affinity to substrates in the glycine cleavage direction than in the glycine synthesis direction [42], which might be the reason why H-protein is not present at a high level in *T. phaeum* when utilizing amino acids. In this study, only very low enzyme activity of GCS could be observed in in vitro enzyme assays (Table 2). This is likely a result of the complexity of the GCS components and the complex series of biochemical reactions. Furthermore, in the cultivation of *T. phaeum* with glycine, we have observed that *T. phaeum* could not grow stably in higher glycine concentrations (40 mM). It is possible that the GCS rather serves as a detoxifying enzyme in *T. phaeum*, as glycine may interact negatively with various cell functions [31]. Earlier studies have determined comparably low activities in the milliunit range for GCS in *P. acidaminophilum* [15]. However, the glycine tolerance limit of *T. phaeum* is still unknown.

In contrast to GCS, comparably high activities of serine dehydratase were present in *T. phaeum* during axenic growth with serine, which suggests that this enzyme might be more relevant than GCS despite its low abundance in the proteome (Table 2, Fig 3). Serine dehydratase and glycine hydroxymethyltransferase are two serine-converting enzymes in *T. phaeum*. The glycine hydroxymethyltransferase was abundant in the proteome, mainly under amino acid-utilizing conditions. On the contrary, serine dehydratase was present at comparably low levels under all conditions tested in this study. However, serine dehydratase was highly active during axenic growth with serine, as judged by in vitro enzyme assays (Table 2). The enzyme activity of serine dehydratase (0.52 U/mg, Table 2) in the assay for axenic cultures with 40 mM serine, for example, corresponded to 87.5% of the total serine-converting enzyme activity (0.64 U/mg, Table 2). It remains unknown which enzyme is more active in vivo, as both enzyme activity measurement as well as proteome analysis are testing a rather unnatural system of a protein mixture in vitro, which only distantly reflects the situation in the cell. Yet, despite the low abundance in the proteome, the enzyme assays indicated significantly higher serine dehydratase activity, and we find it justified to conclude that, at least during growth with serine, this enzyme is more relevant than the combined activity of glycine hydroxymethyltransferase and the GCS. However, the presence of both enzyme systems in parallel (i.e., glycine hydroxymethyltransferase and GCS versus serine dehydratase) may indicate that *T. phaeum* is able to fine-tune its metabolism between dissimilation and assimilation. On one hand, serine dehydratase converts serine to pyruvate and ammonia. Pyruvate is then oxidized to acetyl-CoA by NAD^+^-dependent pyruvate dehydrogenase (Tph_c19640 and Tph_c19650) identified in the proteome (S13 Fig). Acetyl-CoA can be converted to acetate through phosphate acetyltransferase and acetate kinase, which yields ATP. Alternatively, pyruvate may be used for the synthesis of other amino acids, which would save metabolic labor for the de novo synthesis of building blocks from CO_2_ [43–45]. On the other hand, glycine hydroxymethyltransferase and the GCS may be more important for the production of methylene-THF and, consequently, the C1-metabolism. Yet, both enzyme systems produce NADH and no ferredoxin, which raises the question of how electrons are transferred to the earlier observed NAD^+^-independent methylene-THF reductase or to formate dehydrogenase and hydrogenase [7,8]. Electrons derived from NADH oxidation could be transferred to an intermediate carrier, possibly a quinone, and then to methylene-THF reduction. With anthraquinone-2,6-disulfonate (AQDS) as artificial electron acceptor, an average activity of 0.095 U/mg of NADH:AQDS oxidoreductase was previously detected in soluble fractions of *T. phaeum* grown in syntrophic cultures with acetate [8]. The activity of AQDS-dependent methyl-THF oxidation was 0.024 U/mg in average and it was concluded that electron transfer from NADH to methylene-THF is theoretically possible, though indirect [8]. Furthermore, in this study, we also observe MTHFR activity with NADH as electron donor mainly in the soluble fraction (0.022 U/mg), and NADH:AQDS oxidoreductase activity in both soluble and membrane fractions of *T. phaeum* grown in axenic cultures with serine. Yet, the activity in the membrane fraction is more than five times higher compared to the soluble fraction. It is therefore possible that the electron transfer between NADH oxidation and methylene-THF reduction is catalyzed by a membrane-bound enzyme. In addition, proton translocation across the membrane via Ech hydrogenase may play a role in energy conservation as observed in the thermophile *M. thermoacetica* [46, Fig 1C]. Three of the five Ech hydrogenase subunits were identified in the proteome of cells grown with serine, glycine, acetate or methanol, and one of the subunits (Ech hydrogenase subunit E) was above average with the highest area value in glycine syntrophic and serine syntrophic conditions (S18 Fig). The results presented in this study demonstrate that two different peripheral enzyme systems for glycine or serine degradation are produced and active in *T. phaeum*, with serine dehydratase being the most active enzyme despite its low abundance in the proteome. However, the activity of glycine (serine) hydroxymethyltransferase in the glycine consumption direction was not determined in this study. It remains possible that glycine could be converted to pyruvate via glycine (serine) hydroxymethyltransferase and serine dehydratase, and finally converted to acetate. The contribution of this branch of the pathway to glycine degradation is still unclear. Moreover, whether this branch competes with the WLP for methylene-THF remains to be determined.

We have analyzed the function of glycine- and serine-converting enzyme systems that are present in *T. phaeum*. It appears that the previously postulated alternative pathway of syntrophic acetate oxidation via pyruvate, serine, and glycine may not be relevant in *T. phaeum,* as the genes for serine dehydratase, serine hydroxymethyltransferase, and the GCS are expressed during syntrophic acetate oxidation only at low levels [8,9]. Instead, the glycine- and serine-converting enzyme systems appear to be produced mainly when *T. phaeum* grows with amino acids, which is consistent with the earlier finding that the transcription of the glycine cleavage system genes is enhanced by glycine riboswitches in *Streptomyces griseus* [31]. We therefore conclude a pathway for glycine and serine degradation in *T. phaeum* that deviates from the established models in *P. acidaminophilum* and other mesophilic microbes, relying solely on the GCS and the WLP. In the absence of a glycine-reducing enzyme such as glycine reductase, glycine or serine is converted to methylene-THF, NADH, CO_2_, and NH_3_. Methylene-THF, in turn, is disproportionated to methyl-THF and methenyl-THF by methylene-THF reductase and methylene-THF dehydrogenase, respectively (Fig 1C). Moreover, *P. acidaminophilum* produces hydrogen during growth with glycine, whereas *T. phaeum*, in contrast, most likely internally re-oxidizes electron carriers through the WLP to produce acetate [15]. We therefore postulate stoichiometries for the axenic degradation of glycine, serine and threonine by *T. phaeum*, which yield acetate, ammonia, and carbon dioxide, but no hydrogen (Eq 1, 2, and 3). Under syntrophic conditions, degradation of glycine or serine occurs through partly the same central metabolic routes as under axenic conditions, yet with different stoichiometries and with different peripheral enzymes for releasing hydrogen and/or formate to the methanogenic partner (Fig 1C). For instance, degradation of 4 mols of serine under axenic conditions yields 5 acetate (3 from the WLP and 2 from pyruvate oxidation), while under syntrophic conditions, presumably only 2 acetate are formed exclusively from pyruvate oxidation (Fig 1C). This is due to the assumption, that under syntrophic conditions the reductive branch of the WLP is not needed for releasing excess electrons from NADH. Instead, electrons may be transferred to the methanogenic partner as formate or hydrogen by FTHFL, Ech-hydrogenase, or membrane-bound hydrogenase or formate dehydrogenase (Fig 1C, Eq. 5). Analogously, glycine degradation under axenic conditions yields more acetate (3 mols acetate per 4 mols glycine) compared to syntrophic conditions (2 mols acetate per 4 mols glycine), as under axenic conditions the reductive branch of the WLP serves as electron sink for re-oxidizing NADH (Fig 1C, upward-facing arrows of the WLP, Eq. 4).

During growth with threonine, we observed growth in both syntrophic (S14 Fig) and axenic cultures (S17 Fig). However, growth was unstable, especially in axenic culture. Occasionally, no growth occurred after inoculation into fresh media. We suggest that more acetate should be produced in axenic culture per mole threonine compared to glycine or serine (Eq. 3). However, due to the poor growth and incomplete substrate utilization of *T. phaeum* in axenic culture with threonine, we were unable to demonstrate this in growth experiments. During syntrophic growth, substrate consumption primarily occurred during the long stationary phase, with minimal OD_600_ increase and delayed methane production (methane was detected after 190 hours of incubation and later accumulated to about 1 mM in the incomplete growth with 10 mM threonine as substrate). According to proteome analysis, threonine synthase, which catalyzes the conversion between L-threonine and o-phospho-L-homoserine, was moderately abundant (S7 Fig). Although this enzyme is possibly involved in glycine, serine, and threonine metabolism, it is unlikely that it directly links threonine to the amino acid degradation pathway we are suggesting in this study. No L-threonine aldolase was found, which could directly split threonine into glycine and acetaldehyde, like in *C. ljungdahlii* [47]. Moreover, no threonine dehydrogenase was identified, which could indirectly produce glycine from threonine [48]. The highly upregulated ketol-acid reductoisomerase catalyzes the synthesis of branched amino acids [49]. However, it is unclear why this enzyme was overabundant during growth with threonine. Possibly, the degradation of threonine may involve pathways that convert it to a branched amino acid, different from the one we propose here.

As shown by proteome analysis, membrane-bound formate dehydrogenase genes were abundant under syntrophic growth conditions, including the syntrophic culture with threonine (S9 - S11 Figs). This indicates that *T. phaeum* is likely using both formate and hydrogen for interspecies electron transfer [50,51] also during syntrophic degradation of serine, glycine and threonine.

## Conclusion

Besides various C1 and C2 compounds such as methanol, ethanol, and ethanolamine, the thermophilic acetogen *Thermacetogenium phaeum* grows with glycine, serine, and threonine as sole energy sources. Potential serine- and glycine-converting enzyme systems, such as serine dehydratase, glycine hydroxymethyltransferase, glycine cleavage system and pyruvate dehydrogenase, were identified in the proteome and characterized by in vitro enzyme assays. Activities of a key Wood-Ljungdahl pathway enzyme, methylene-THF reductase, as well as NADH:acceptor oxidoreductase were identified in in vitro enzyme assays. As genes for glycine reductase are missing in the genome of *T. phaeum*, we describe here modified pathways of glycine or serine degradation that deviate from established model systems: a glycine cleavage system is mainly responsible for the degradation of glycine, which produces methylene-THF. Methylene-THF is disproportionated to methenyl-THF and methyl-THF. On the other hand, serine degradation apparently rather occurs through serine dehydratase instead of glycine hydroxymethyltransferase and the glycine cleavage system, even though the abundance of serine dehydratase was comparably low in the proteomes of *T. phaeum*. Despite the fact that *T. phaeum* is mainly known for its ability to oxidize acetate in syntrophic coculture with *Methanothermobacter thermautotrophicus*, surprisingly, *T. phaeum* is unable to shift to syntrophic acetate oxidation once glycine or serine are converted to acetate, i.e., complete mineralization of amino acids is impossible for unknown reasons. Special challenges for thermophilic microbial substrate conversions that are not present in mesophilic systems, such as thermolabile enzymes and metabolic intermediates, distinguish thermophiles from their mesophilic counterparts and may help explain why they undergo pathways that differ from mesophilic systems.

## Supporting information

S1 Fig Growth experiments of *T. phaeum* with different substrates at 55°C or 61°C. A: axenic culture with 10 mM glycine, B: syntrophic culture with 10 mM glycine, C: axenic culture with 40 mM serine, D: syntrophic culture with 40 mM serine, E: syntrophic culture with 40 mM acetate, F: syntrophic culture with 10 mM threonine. All the conditions were investigated in triplicate.
(TIF)

S2 Fig Growth curves of syntrophic cultures of *T. phaeum* and *M. thermautotrophicus* with 40 mM Na-acetate. The effect of addition of varying concentrations of ammonium chloride (NH_4_Cl) is shown.
(TIF)

S3 Fig Serine consumption, pyruvate production and glycine production in serine-converting enzyme assays. A: Comparing serine axenic culture and acetate syntrophic culture. B: Comparing serine axenic culture and serine syntrophic culture.
(TIF)

S4 Fig Glycine consumption over time in glycine cleavage system enzyme assays. (TIF)

S5 Fig Proteome data of serine dehydratase and glycine hydroxymethyltransferase obtained during axenic or syntrophic growth with non-amino acid substrates (methanol, ethanolamine, ethanol or acetate). Samples from different growth conditions were labelled with different TMT-labels and then mixed for further preparation and analysis. Shown are non-normalized area values compared to the area value of housekeeping proteins (ATPase, RNA-polymerase and molecular chaperone DnaK). Locus tags are shown without “Tph_” prefix (e.g., Tph_c17140 is shown as c17140). Data were obtained from quadruplicate measurements.
(TIF)

S6 Fig Proteome data of glycine cleavage system obtained during axenic or syntrophic growth with non-amino acid substrates (methanol, ethanolamine, ethanol or acetate). Samples from different growth conditions were labelled with different TMT-labels and then mixed for further preparation and analysis. Shown are non-normalized area values compared to the area value of housekeeping proteins (ATPase, RNA-polymerase and molecular chaperone DnaK). Locus tags are shown without “Tph_” prefix (e.g., Tph_c17170 is shown as c17170). Data were obtained from quadruplicate measurements.
(TIF)

S7 Fig Proteome data of genes potentially involved in threonine metabolism obtained during axenic or syntrophic growth with glycine, serine or threonine. Samples from different growth conditions were labelled with different TMT-labels and then mixed for further preparation and analysis. Shown are non-normalized area values compared to the area value of housekeeping proteins (ATPase, RNA-polymerase and molecular chaperone DnaK). Locus tags are shown without “Tph_” prefix (e.g., Tph_c19850 is shown as c19850). Data were obtained from triplicate measurements.
(TIF)

S8 Fig Volcano plots of threonine syntrophic conditions compared to other growth conditions with amino acids as substrates. A: comparison with serine axenic culture, B: comparison with serine syntrophic culture, C: comparison with glycine syntrophic culture, D: comparison with glycine axenic culture, E: comparison with threonine axenic culture. Membrane-bound formate dehydrogenase (fdhA1, fdhA2, fdxH, fdh subunit), quinone synthesis genes (ubiD1, ubiX), ketol-acid reductoisomerase (ilvC) are highlighted in black circles. Log2fold change > 1 or <−1, p-value<0.05 were selected as up- or down-regulated genes. Data were obtained from triplicate measurements.
(TIF)

S9 Fig Proteome data of membrane-bound formate dehydrogenase and the adjacent gene cluster with quinone synthesis genes obtained during axenic or syntrophic growth with glycine, serine or threonine. Samples from different growth conditions were labelled with different TMT-labels and then mixed for further preparation and analysis. Shown are non-normalized area values compared to the area value of housekeeping proteins (ATPase, RNA-polymerase and molecular chaperone DnaK). Locus tags are shown without “Tph_” prefix (e.g., Tph_c15370 is shown as c15370). Data were obtained from triplicate measurements.
(TIF)

S10 Fig Proteome data of membrane-bound formate dehydrogenase and the adjacent gene cluster with quinone synthesis genes obtained during axenic or syntrophic growth with non-amino acid substrates (methanol, ethanol, ethanolamine or acetate). Samples from different growth conditions were labelled with different TMT-labels and then mixed for further preparation and analysis. Shown are non-normalized area values compared to the area value of housekeeping proteins (ATPase, RNA-polymerase and molecular chaperone DnaK). Locus tags are shown without “Tph_” prefix (e.g., Tph_c15370 is shown as c15370). Data were obtained from quadruplicate measurements.
(TIF)

S11 Fig Proteome data of membrane-bound formate dehydrogenase and the adjacent gene cluster with quinone synthesis genes obtained during axenic or syntrophic growth with glycine, serine, acetate or methanol. Samples from different growth conditions were labelled with different TMT-labels and then mixed for further preparation and analysis. Shown are non-normalized area values compared to the area value of housekeeping proteins (ATPase, RNA-polymerase and molecular chaperone DnaK). Locus tags are shown without “Tph_” prefix (e.g., Tph_c15370 is shown as c15370). Data were obtained from triplicate measurements.
(TIF)

S12 Fig Proteome data of Wood-Ljungdahl pathway genes obtained during axenic or syntrophic growth with non-amino acid substrates (methanol, ethanolamine, acetate or ethanol). Samples from different growth conditions were labelled with different TMT-labels and then mixed for further preparation and analysis. Shown are non-normalized area values compared to the area value of housekeeping proteins (ATPase, RNA-polymerase and molecular chaperone DnaK). Locus tags are shown without “Tph_” prefix (e.g., Tph_c08280 is shown as c08280). Data were obtained from quadruplicate measurements.
(TIF)

S13 Fig Proteome data of pyruvate dehydrogenase genes obtained during axenic or syntrophic growth with glycine, serine, acetate or methanol. Samples from different growth conditions were labelled with different TMT-labels and then mixed for further preparation and analysis. Shown are non-normalized area values compared to the area value of housekeeping proteins (ATPase, RNA-polymerase and molecular chaperone DnaK). Locus tags are shown without “Tph_” prefix (e.g., Tph_c19620 is shown as c19620). Data were obtained from triplicate measurements.
(TIF)

S14 Fig Growth of *T. phaeum* in syntrophic culture with threonine as substrate incubated at 55°C. Data were obtained from triplicate measurements.
(TIF)

S15 Fig Diagram of pyruvate production in serine dehydratase enzyme assays. (TIF)

S16 Fig Function of temperature and growth rate of *T. phaeum* cultures with amino acids or acetate. (TIF)

S17 Fig Growth of *T. phaeum* in axenic culture with threonine as substrate, incubated at 55°C. Data were obtained from triplicate measurements.
(TIF)

S18 Fig Proteome data of Ech dehydrogenase subunits obtained during axenic or syntrophic growth with glycine, serine, acetate or methanol. Samples from different growth conditions were labelled with different TMT-labels and then mixed for further preparation and analysis. Shown are non-normalized area values compared to the area value of housekeeping proteins (ATPase, RNA-polymerase and molecular chaperone DnaK). Locus tags are shown without “Tph_” prefix (e.g., Tph_c21360 is shown as c21360). Data were obtained from triplicate measurements.
(TIF)

S1 Table Specific enzyme activities of glycine cleavage system measured in photometric enzyme assays. (PDF)

S1 File Proteomics data, amino acid and non-amino acid area of identified peptides and z-score. (XLSX)

S2 File Proteomics data, non-amino acid area of identified peptides and z-score. (XLSX)

S3 File Proteomics data, amino acid area of identified peptides and z-score. (XLSX)

S4 File Temperature growth tests. (XLSX)
