# Supplementary figures and images for: Amino acid metabolism of the thermophilic acetogen Thermacetogenium phaeum
Source: PLoS One. 2025 Dec 3;20(12):e0336914. doi: 10.1371/journal.pone.0336914 (PMC12674515; doi:10.1371/journal.pone.0336914)

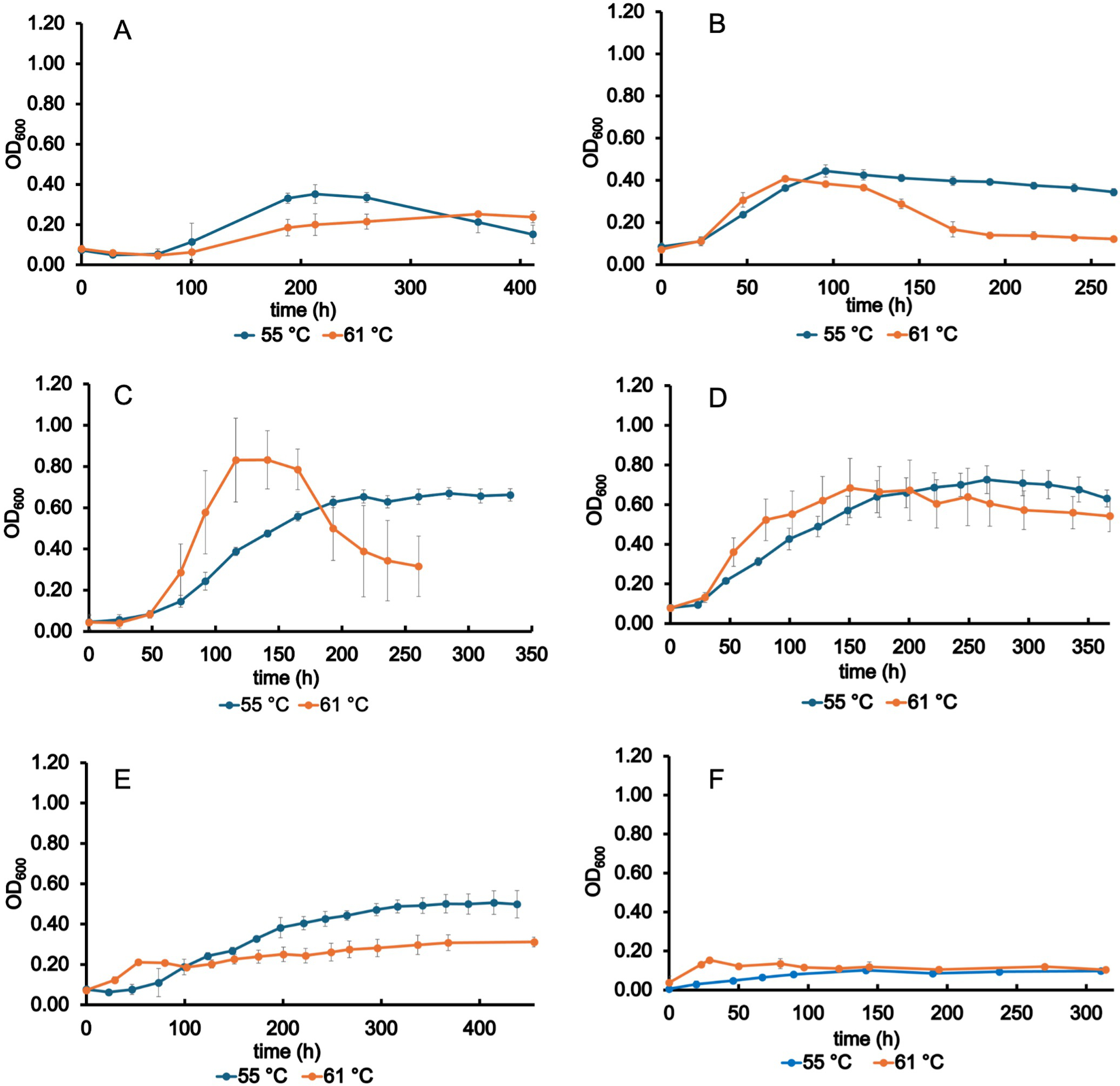

Supplement: S1 Fig — A: axenic culture with 10 mM glycine, B: syntrophic culture with 10 mM glycine, C: axenic culture with 40 mM serine, D: syntrophic culture with 40 mM serine, E: syntrophic culture with 40 mM acetate, F: syntrophic culture with 10 mM threonine. All the conditions were investigated in triplicate. (TIF) [file pone.0336914.s001.tif]

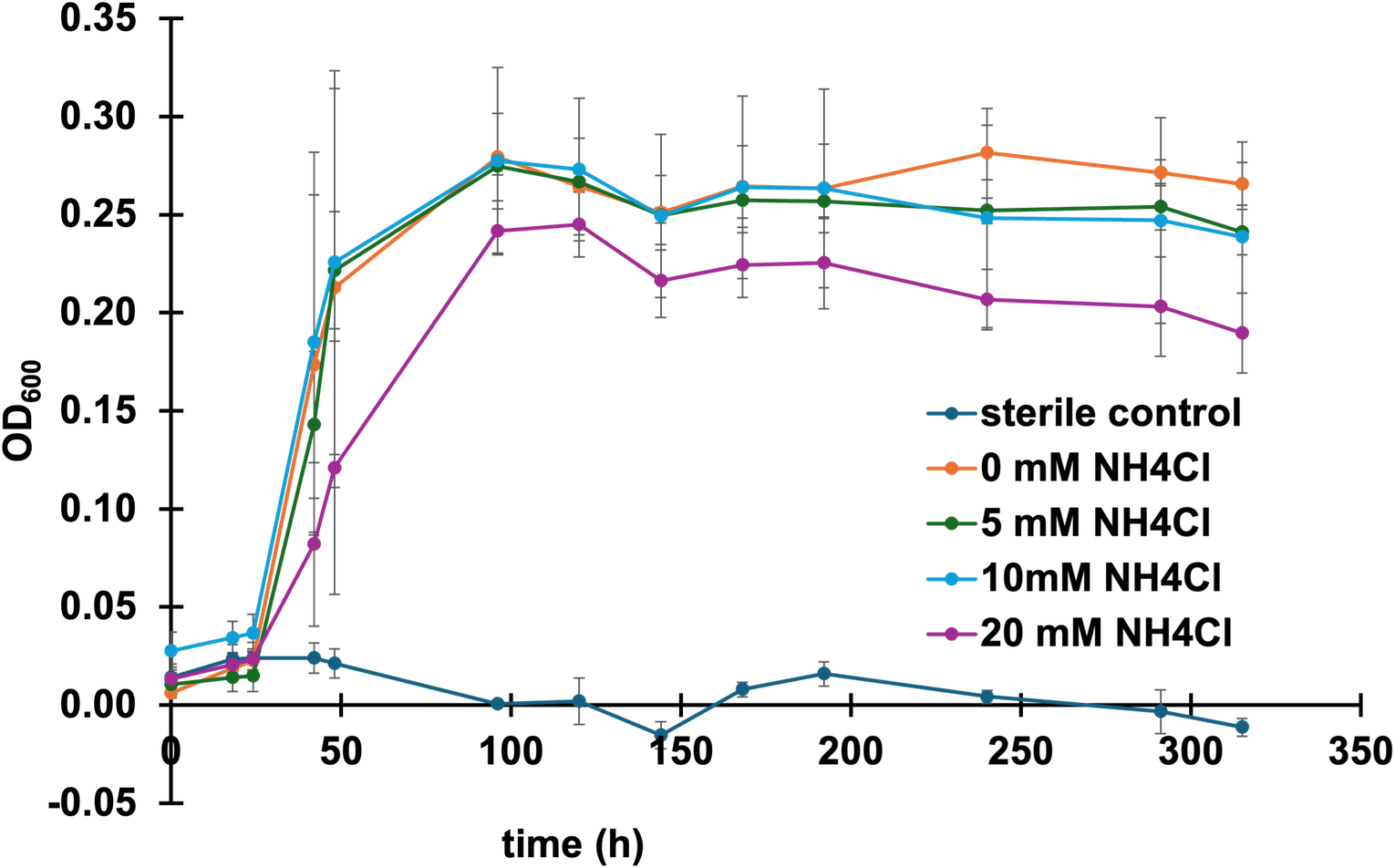

Supplement: S2 Fig — The effect of addition of varying concentrations of ammonium chloride (NH4Cl) is shown. (TIF) [file pone.0336914.s002.tif]

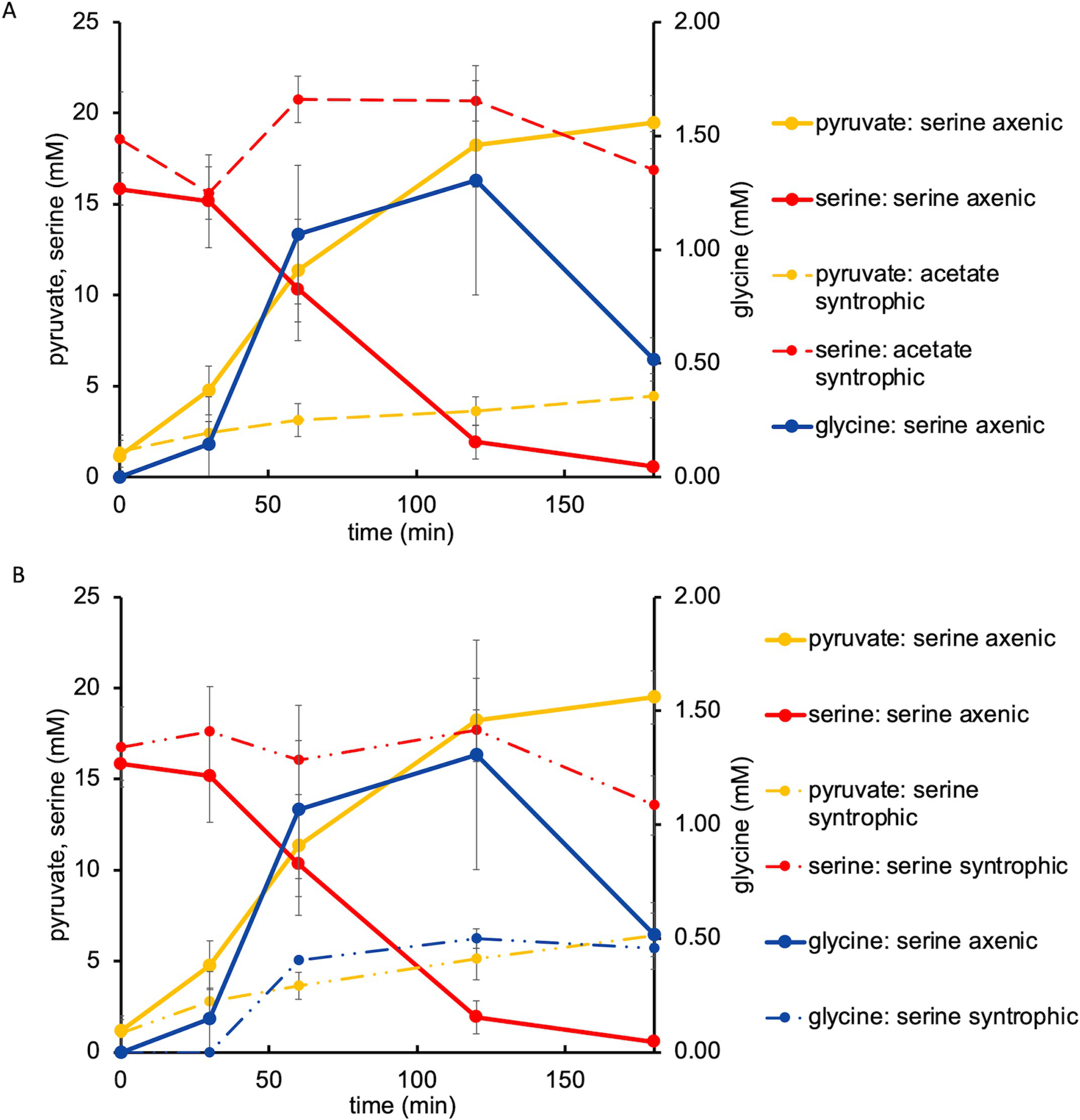

Supplement: S3 Fig — A: Comparing serine axenic culture and acetate syntrophic culture. B: Comparing serine axenic culture and serine syntrophic culture. (TIF) [file pone.0336914.s003.tif]

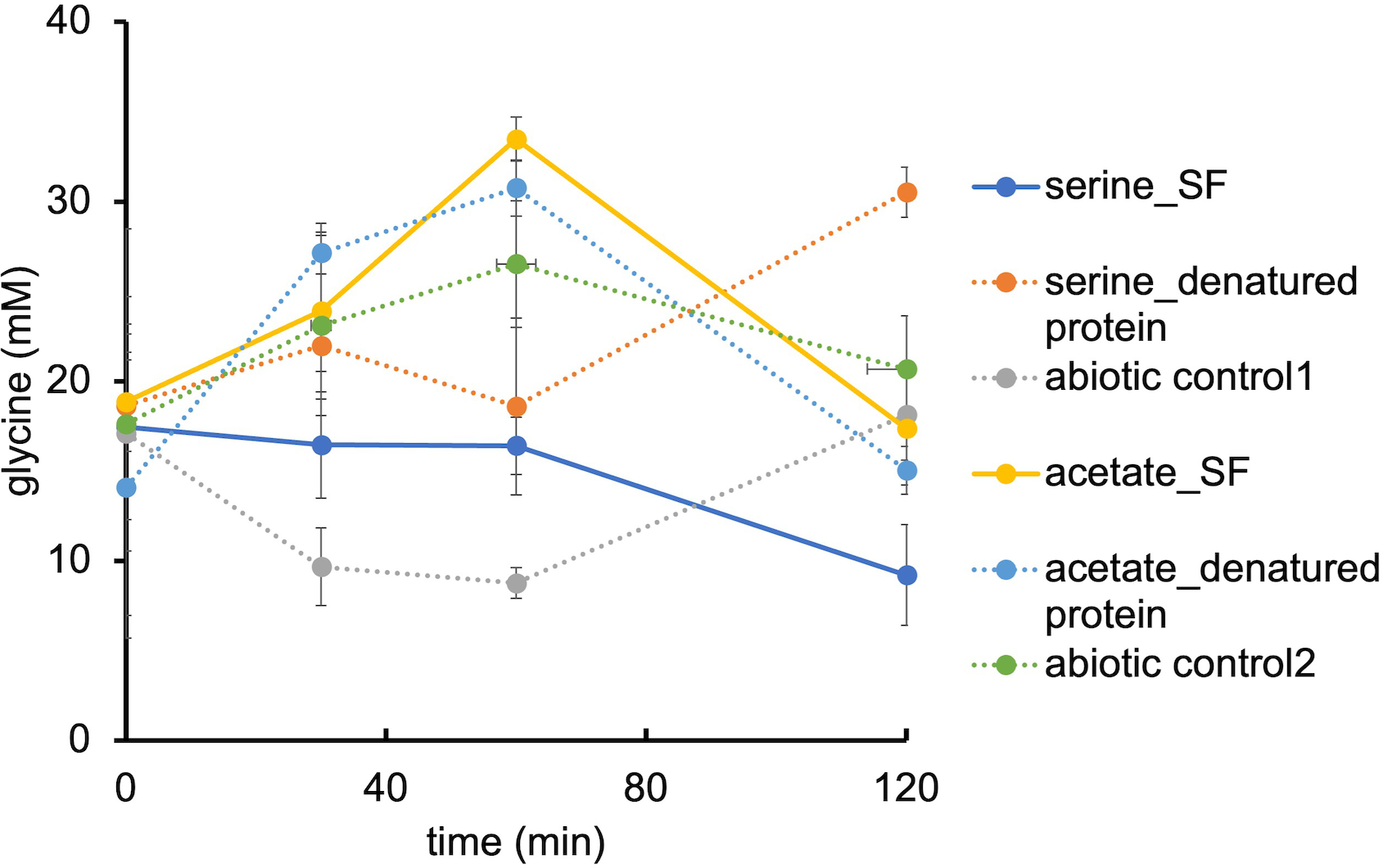

Supplement: S4 Fig — (TIF) [file pone.0336914.s004.tif]

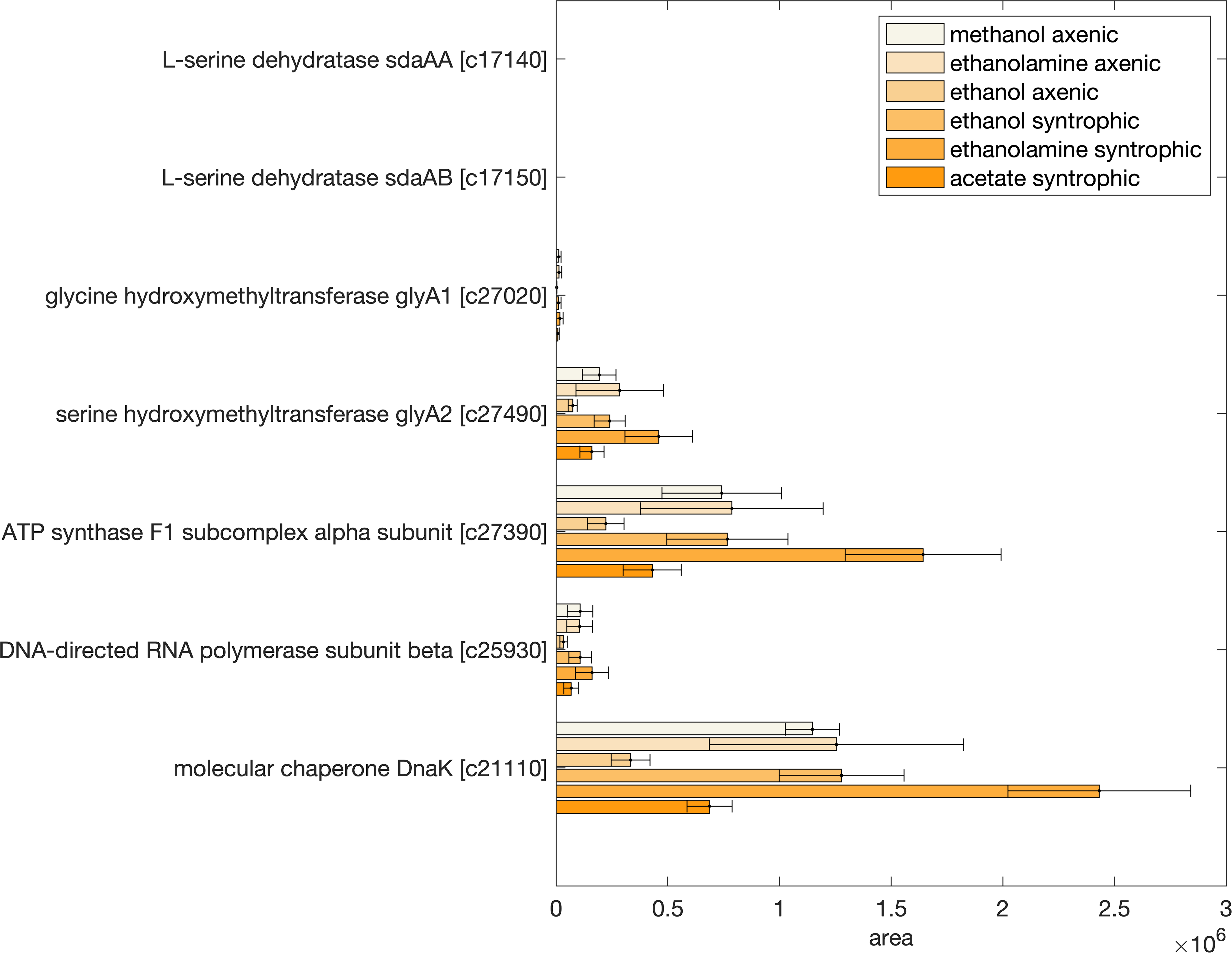

Supplement: S5 Fig — Samples from different growth conditions were labelled with different TMT-labels and then mixed for further preparation and analysis. Shown are non-normalized area values compared to the area value of housekeeping proteins (ATPase, RNA-polymerase and molecular chaperone DnaK). Locus tags are shown without “Tph_” prefix (e.g., Tph_c17140 is shown as c17140). Data were obtained from quadruplicate measurements. (TIF) [file pone.0336914.s005.tif]

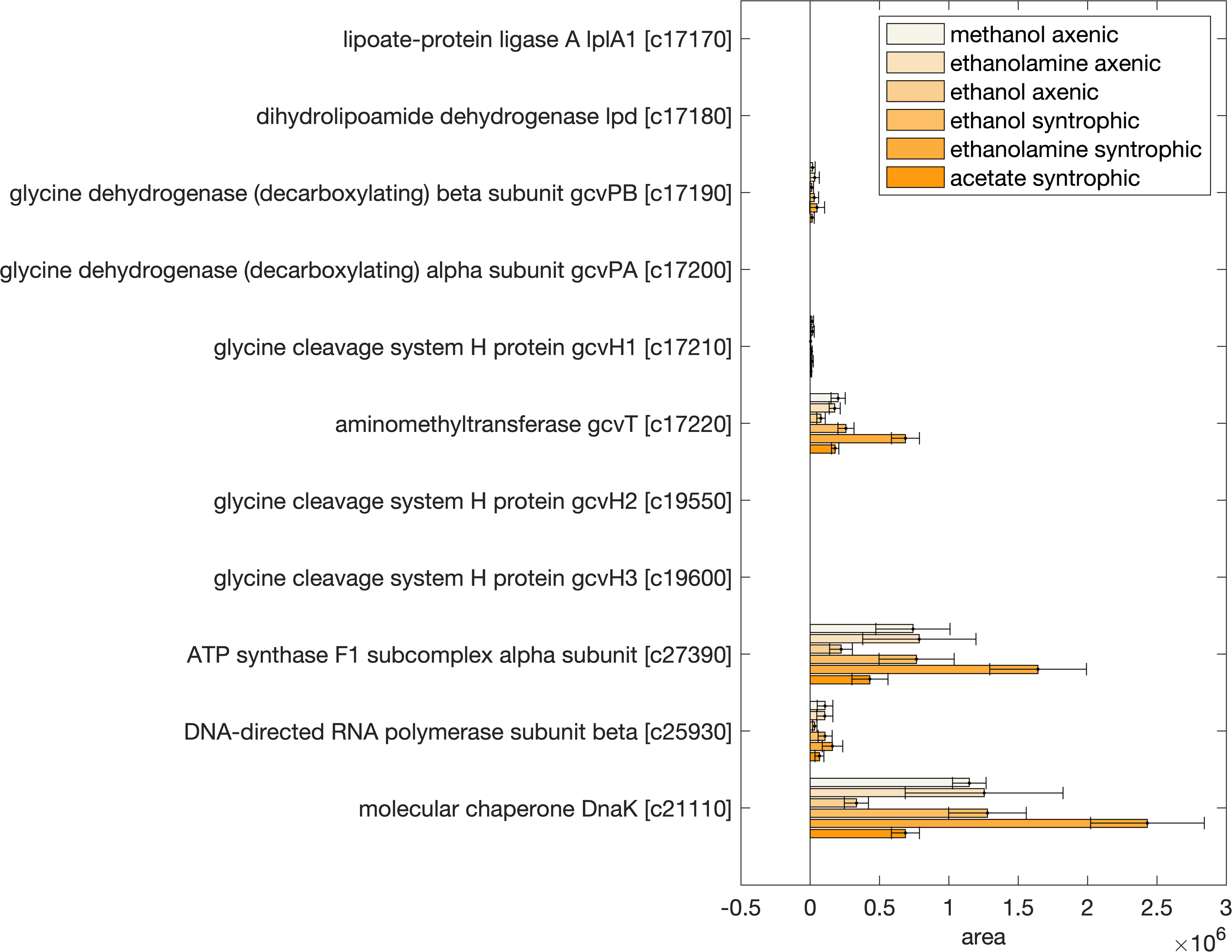

Supplement: S6 Fig — Samples from different growth conditions were labelled with different TMT-labels and then mixed for further preparation and analysis. Shown are non-normalized area values compared to the area value of housekeeping proteins (ATPase, RNA-polymerase and molecular chaperone DnaK). Locus tags are shown without “Tph_” prefix (e.g., Tph_c17170 is shown as c17170). Data were obtained from quadruplicate measurements. (TIF) [file pone.0336914.s006.tif]

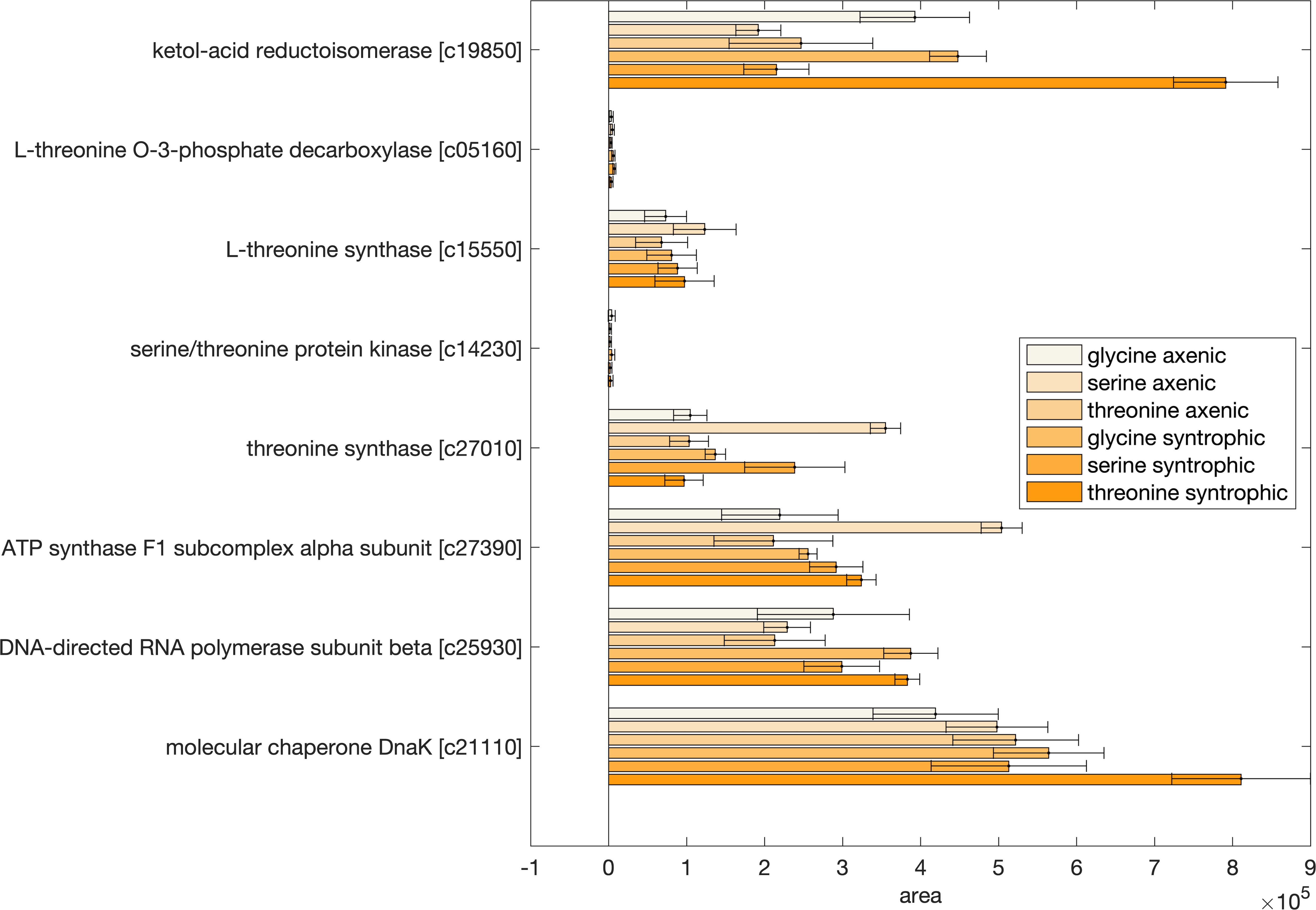

Supplement: S7 Fig — Samples from different growth conditions were labelled with different TMT-labels and then mixed for further preparation and analysis. Shown are non-normalized area values compared to the area value of housekeeping proteins (ATPase, RNA-polymerase and molecular chaperone DnaK). Locus tags are shown without “Tph_” prefix (e.g., Tph_c19850 is shown as c19850). Data were obtained from triplicate measurements. (TIF) [file pone.0336914.s007.tif]

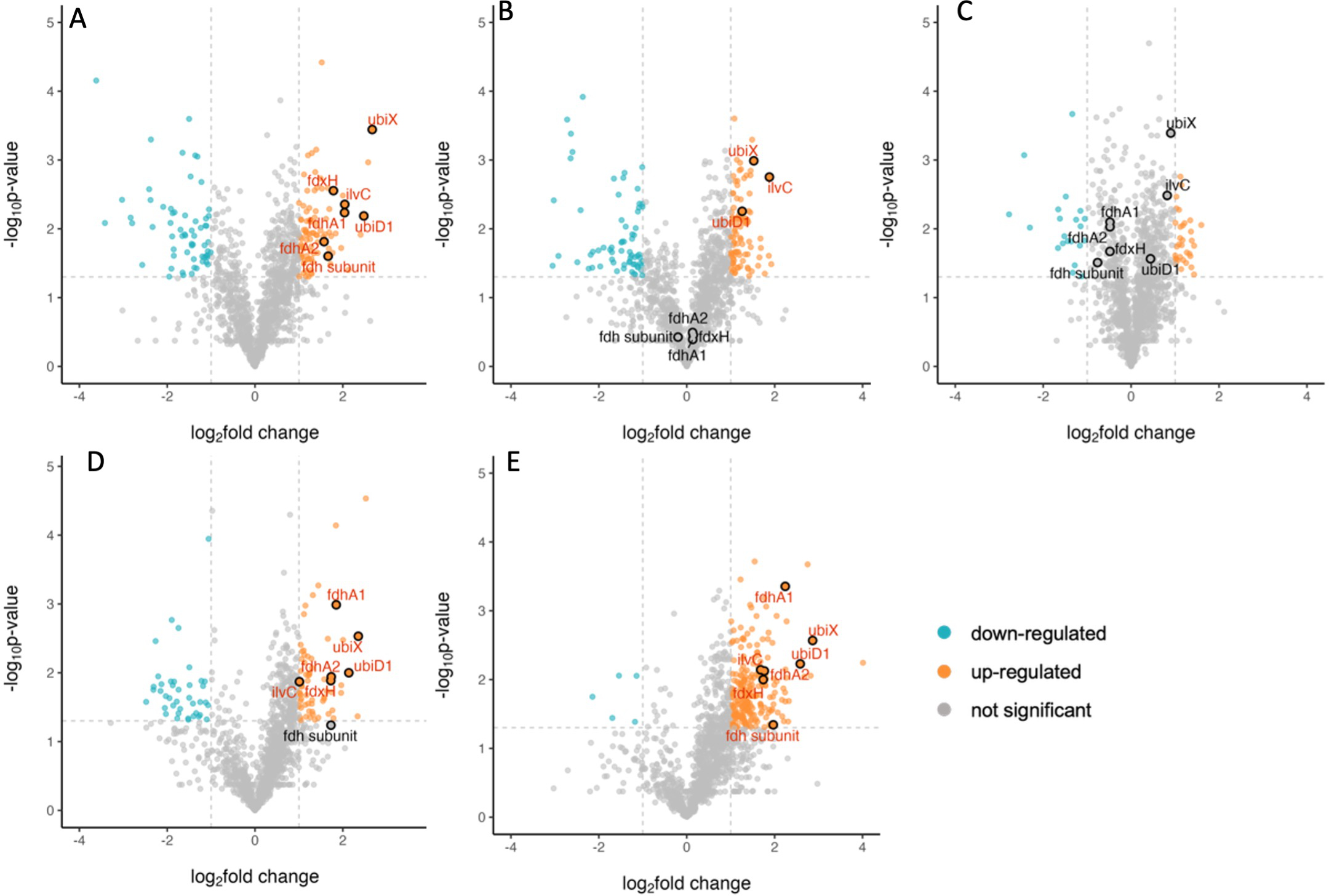

Supplement: S8 Fig — A: comparison with serine axenic culture, B: comparison with serine syntrophic culture, C: comparison with glycine syntrophic culture, D: comparison with glycine axenic culture, E: comparison with threonine axenic culture. Membrane-bound formate dehydrogenase (fdhA1, fdhA2, fdxH, fdh subunit), quinone synthesis genes (ubiD1, ubiX), ketol-acid reductoisomerase (ilvC) are highlighted in black circles. Log2fold change > 1 or <−1, p-value<0.05 were selected as up- or down-regulated genes. Data were obtained from triplicate measurements. (TIF) [file pone.0336914.s008.tif]

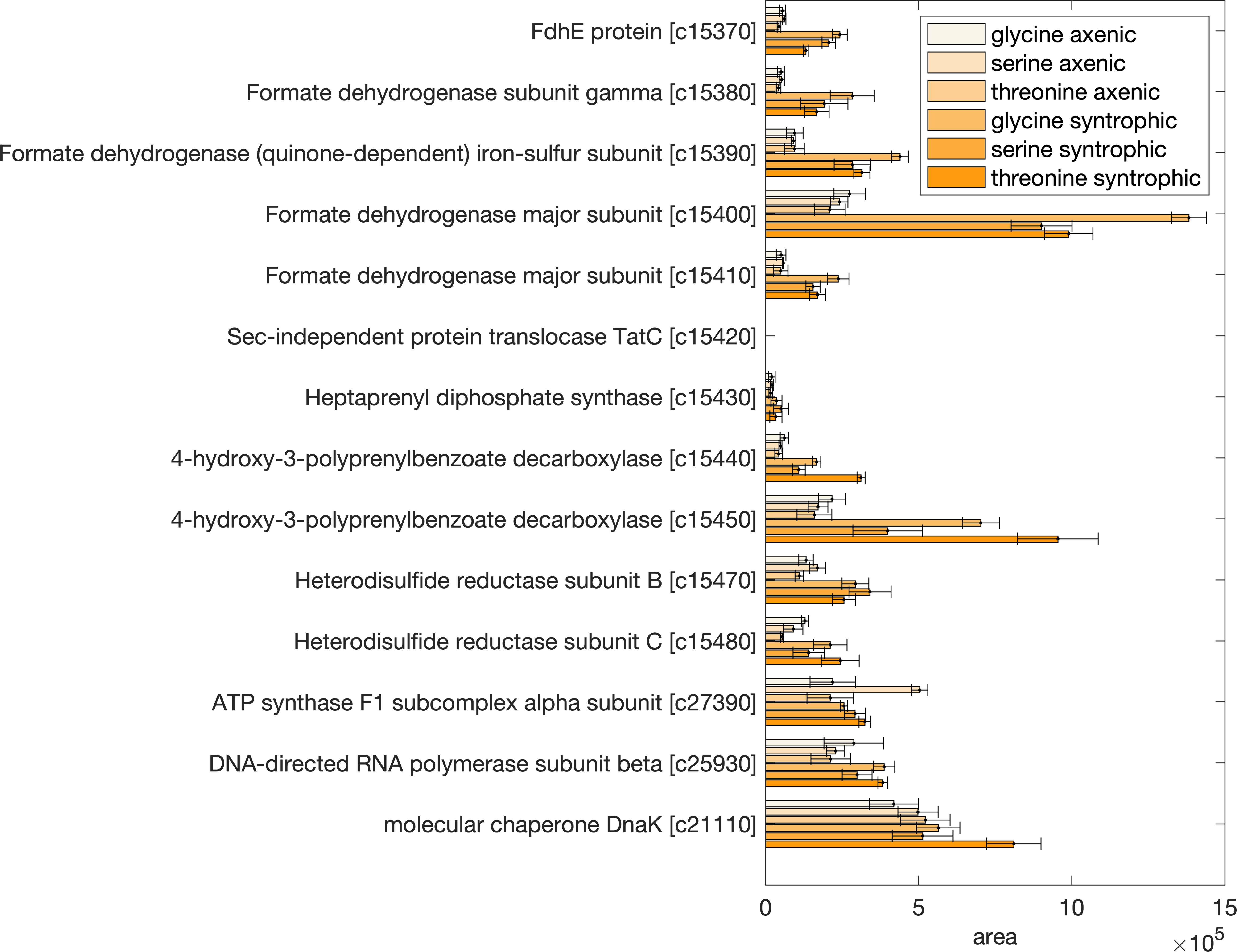

Supplement: S9 Fig — Samples from different growth conditions were labelled with different TMT-labels and then mixed for further preparation and analysis. Shown are non-normalized area values compared to the area value of housekeeping proteins (ATPase, RNA-polymerase and molecular chaperone DnaK). Locus tags are shown without “Tph_” prefix (e.g., Tph_c15370 is shown as c15370). Data were obtained from triplicate measurements. (TIF) [file pone.0336914.s009.tif]

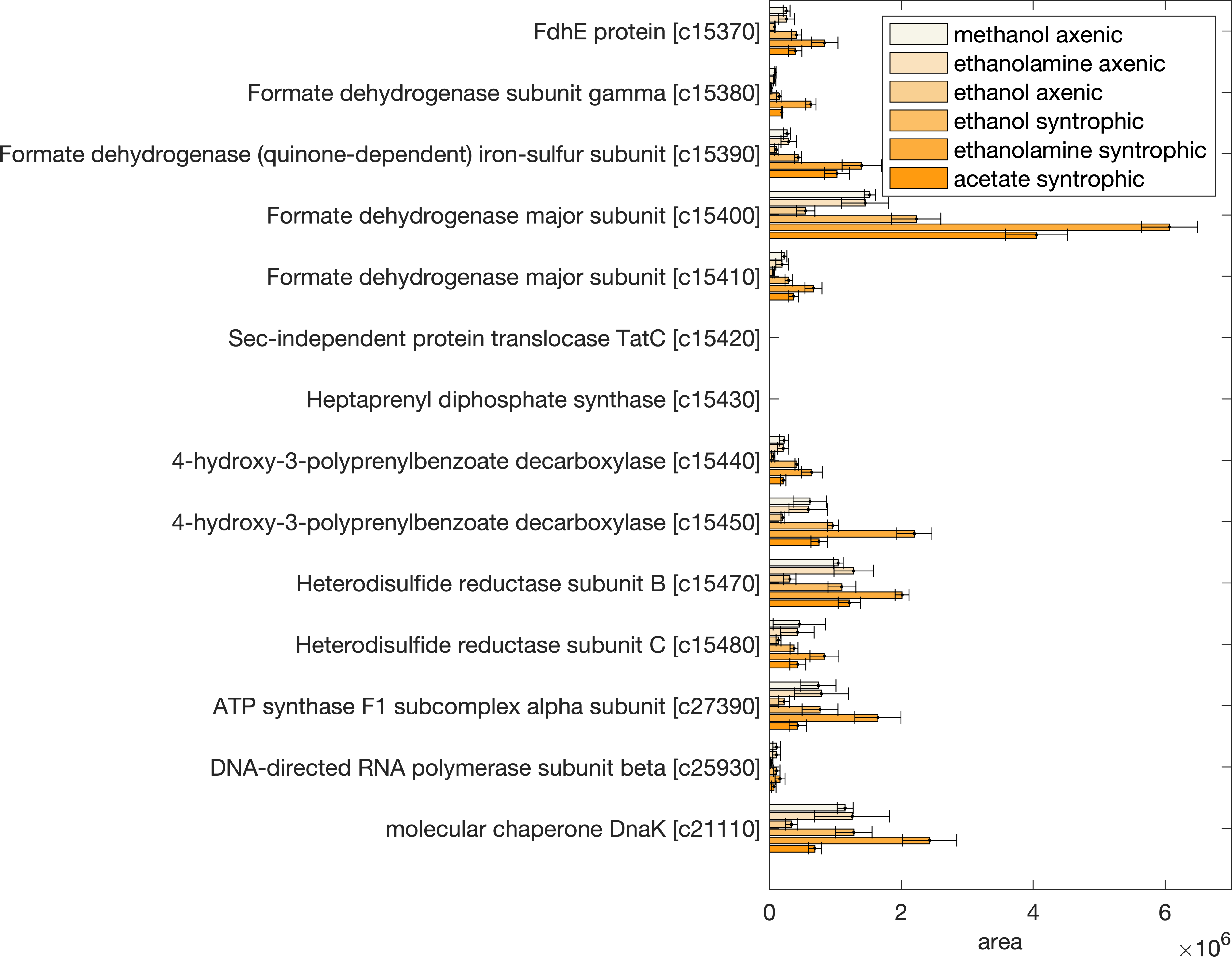

Supplement: S10 Fig — Samples from different growth conditions were labelled with different TMT-labels and then mixed for further preparation and analysis. Shown are non-normalized area values compared to the area value of housekeeping proteins (ATPase, RNA-polymerase and molecular chaperone DnaK). Locus tags are shown without “Tph_” prefix (e.g., Tph_c15370 is shown as c15370). Data were obtained from quadruplicate measurements. (TIF) [file pone.0336914.s010.tif]

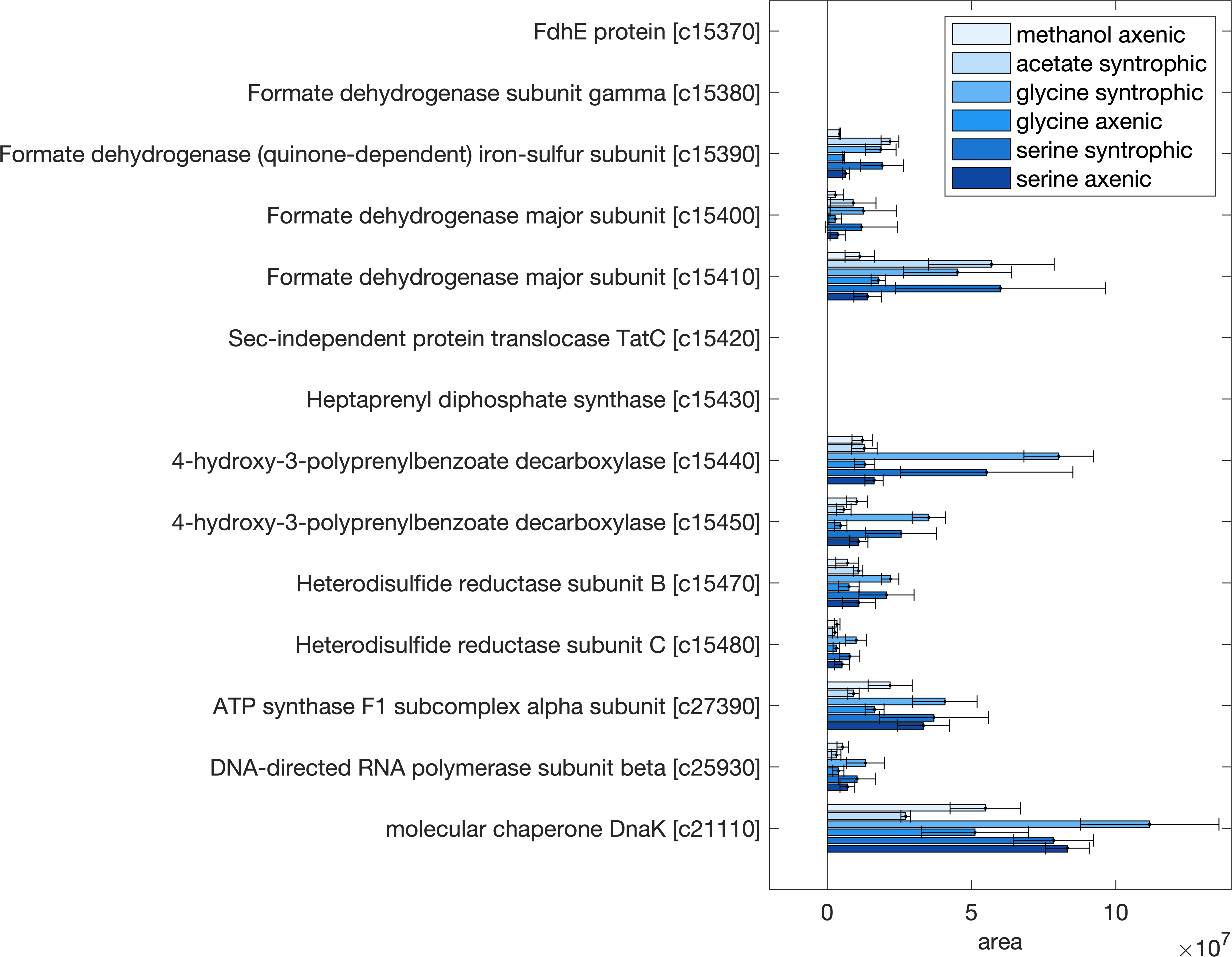

Supplement: S11 Fig — Samples from different growth conditions were labelled with different TMT-labels and then mixed for further preparation and analysis. Shown are non-normalized area values compared to the area value of housekeeping proteins (ATPase, RNA-polymerase and molecular chaperone DnaK). Locus tags are shown without “Tph_” prefix (e.g., Tph_c15370 is shown as c15370). Data were obtained from triplicate measurements. (TIF) [file pone.0336914.s011.tif]

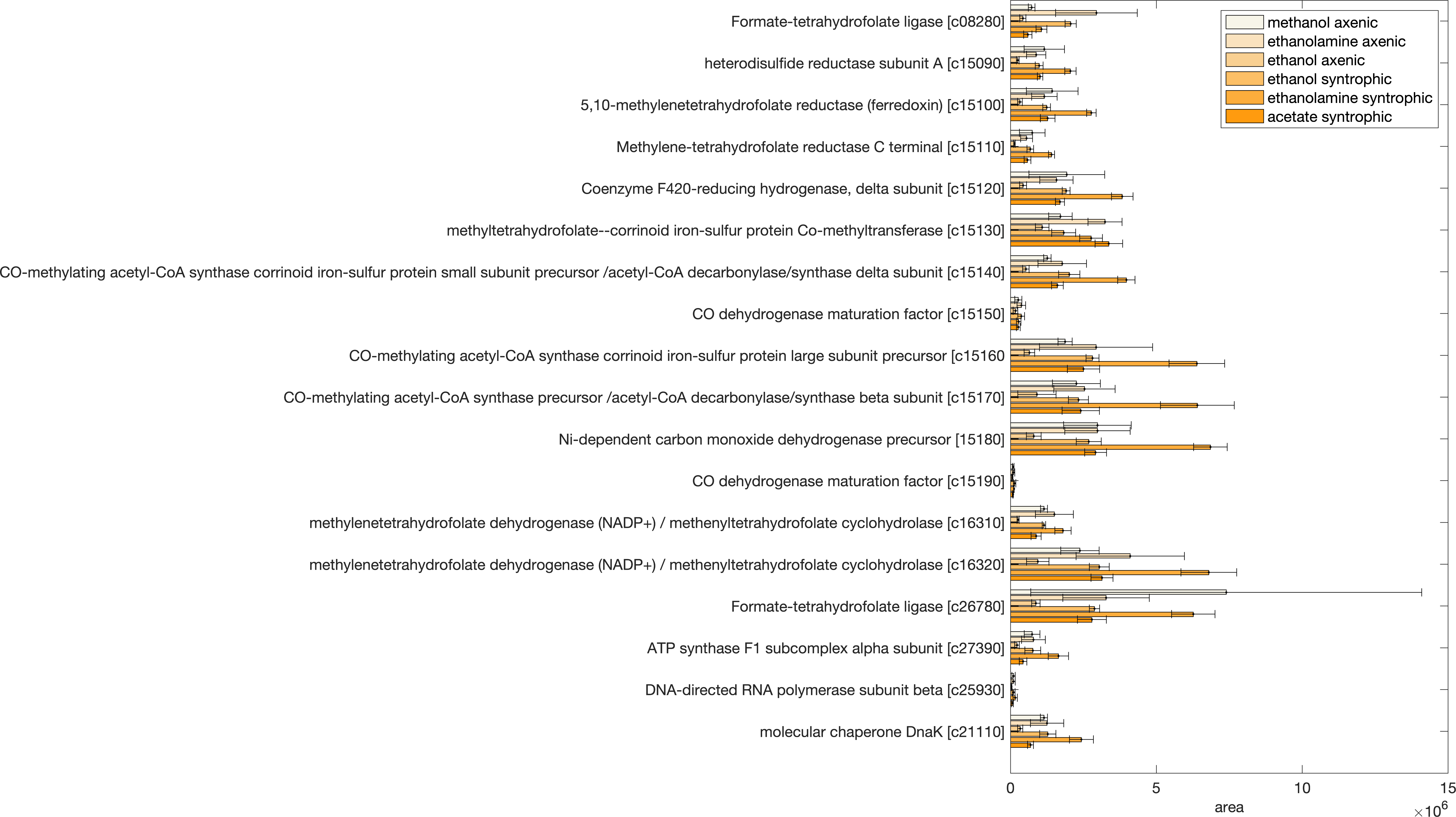

Supplement: S12 Fig — Samples from different growth conditions were labelled with different TMT-labels and then mixed for further preparation and analysis. Shown are non-normalized area values compared to the area value of housekeeping proteins (ATPase, RNA-polymerase and molecular chaperone DnaK). Locus tags are shown without “Tph_” prefix (e.g., Tph_c08280 is shown as c08280). Data were obtained from quadruplicate measurements. (TIF) [file pone.0336914.s012.tif]

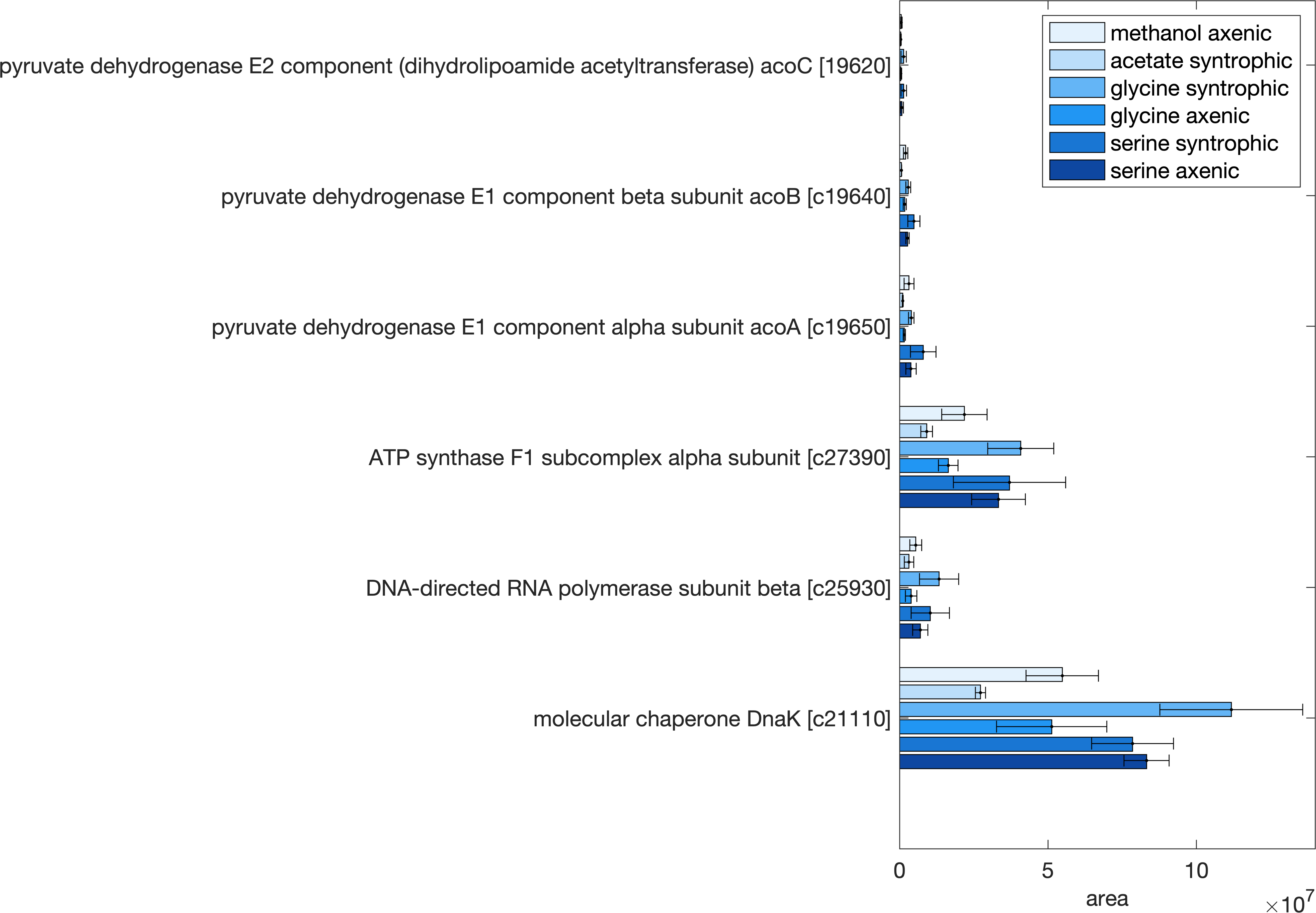

Supplement: S13 Fig — Samples from different growth conditions were labelled with different TMT-labels and then mixed for further preparation and analysis. Shown are non-normalized area values compared to the area value of housekeeping proteins (ATPase, RNA-polymerase and molecular chaperone DnaK). Locus tags are shown without “Tph_” prefix (e.g., Tph_c19620 is shown as c19620). Data were obtained from triplicate measurements. (TIF) [file pone.0336914.s013.tif]

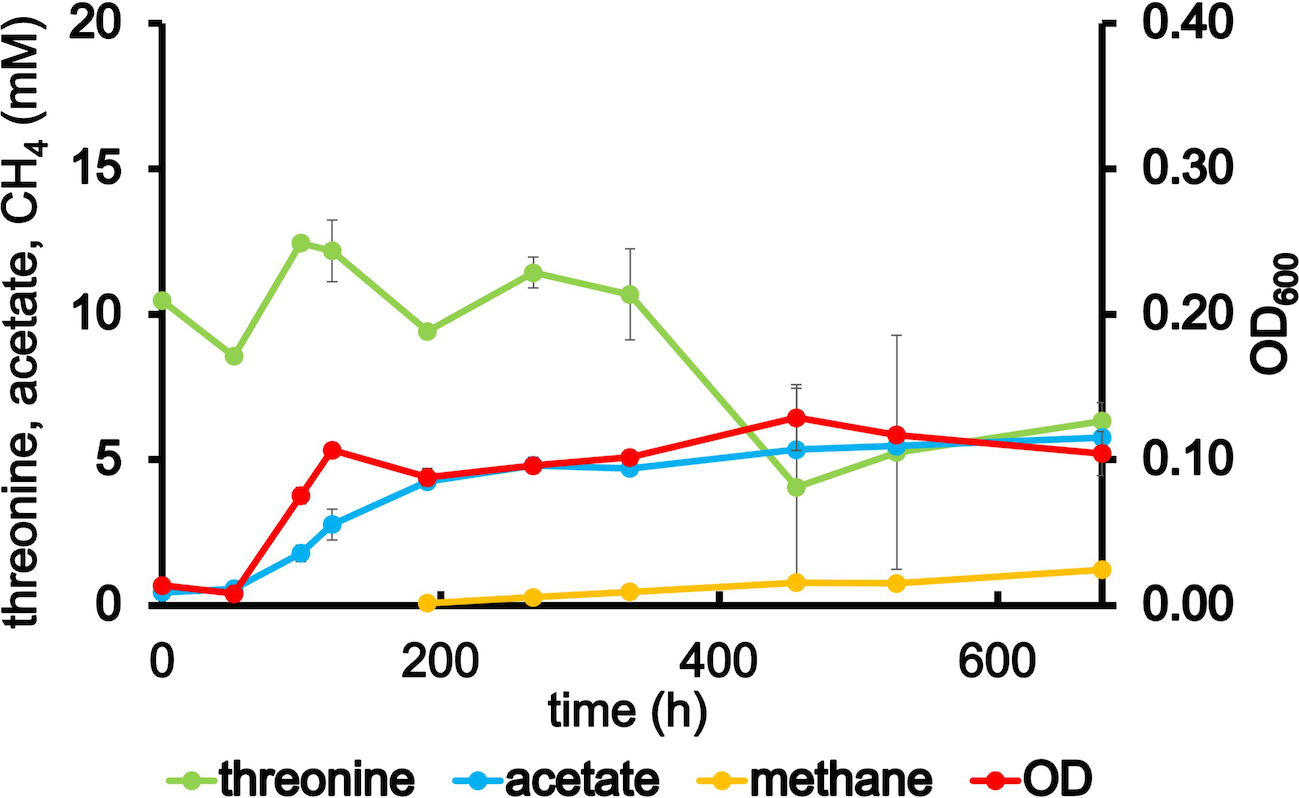

Supplement: S14 Fig — Data were obtained from triplicate measurements. (TIF) [file pone.0336914.s014.tif]

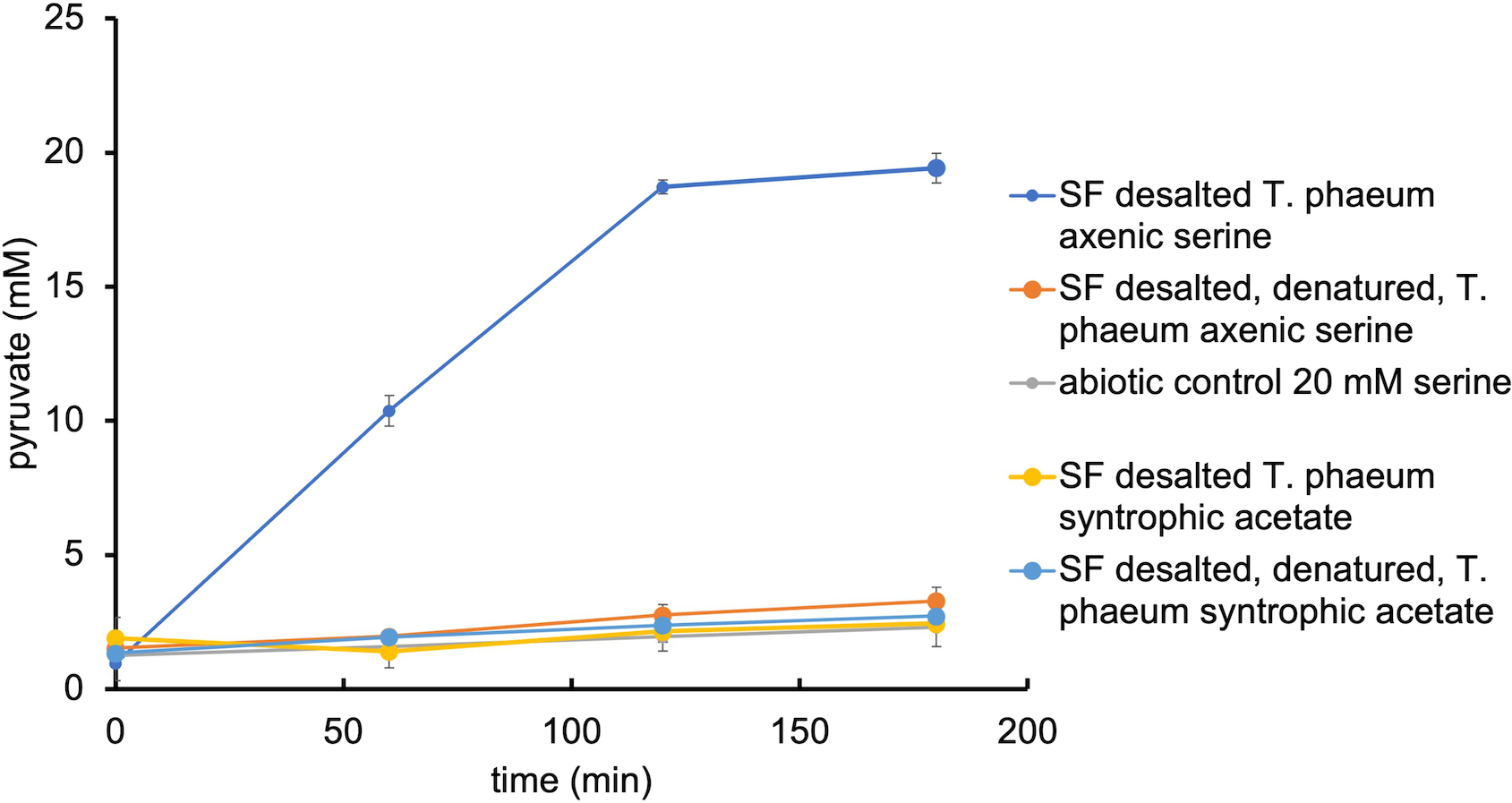

Supplement: S15 Fig — (TIF) [file pone.0336914.s015.tif]

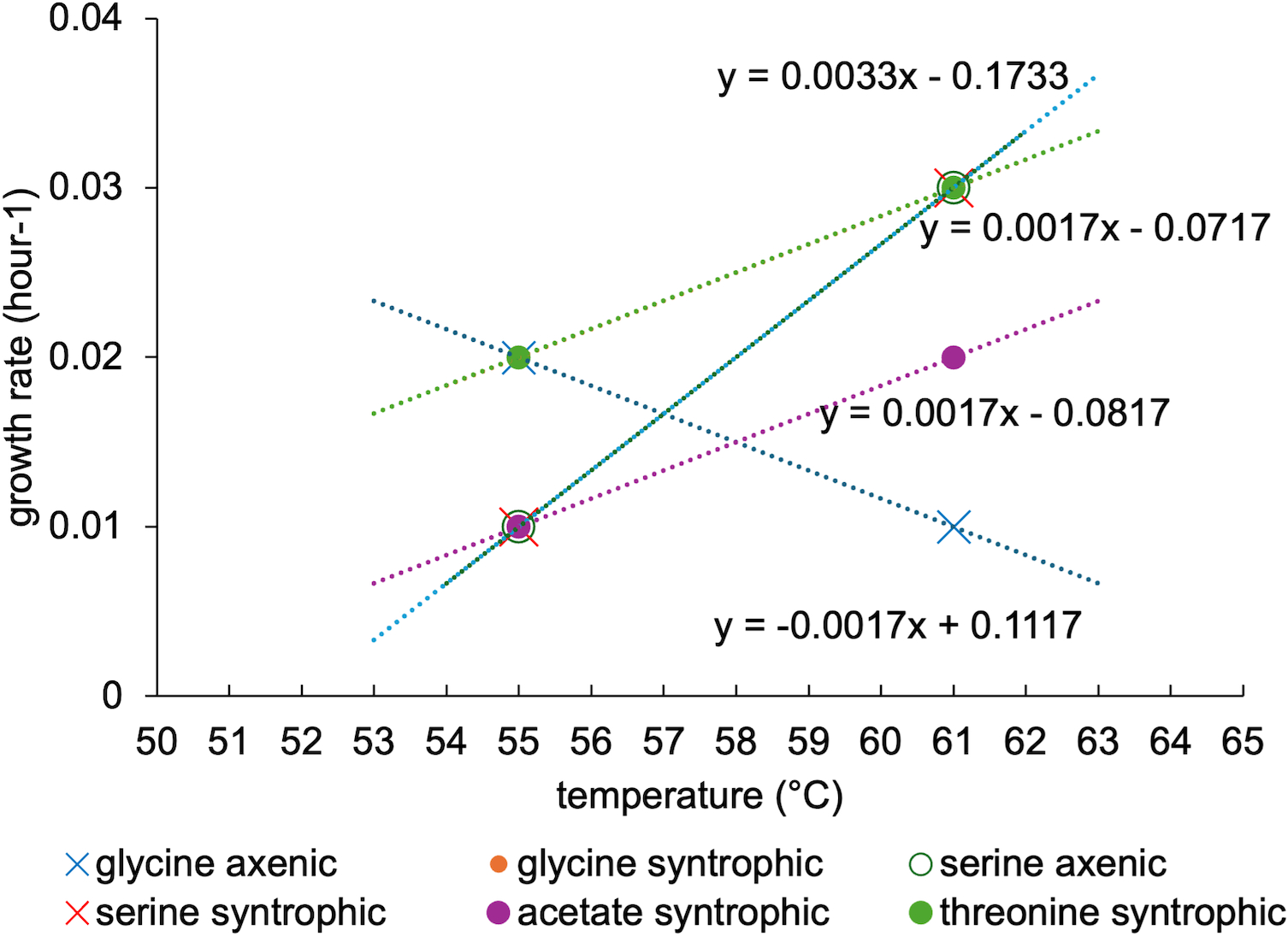

Supplement: S16 Fig — (TIF) [file pone.0336914.s016.tif]

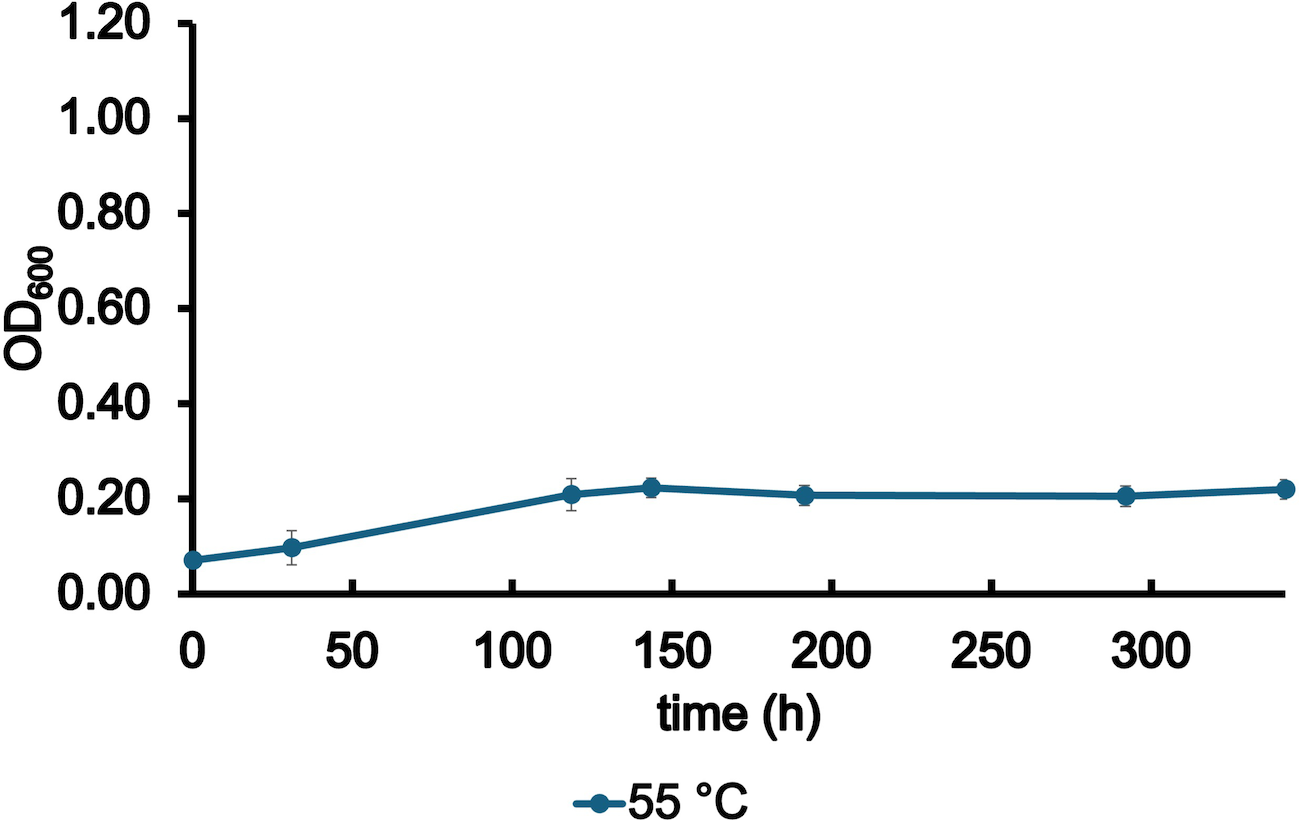

Supplement: S17 Fig — Data were obtained from triplicate measurements. (TIF) [file pone.0336914.s017.tif]

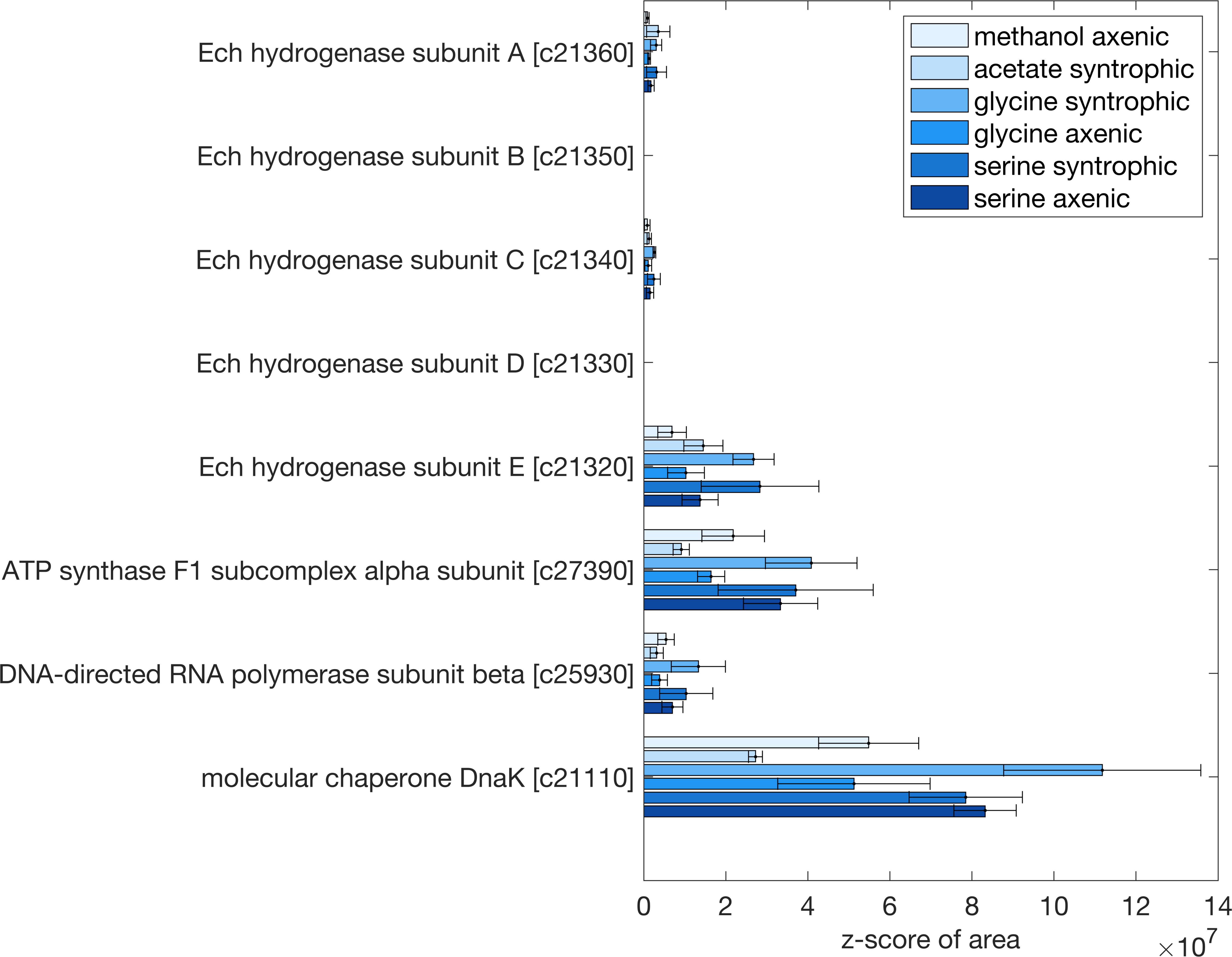

Supplement: S18 Fig — Samples from different growth conditions were labelled with different TMT-labels and then mixed for further preparation and analysis. Shown are non-normalized area values compared to the area value of housekeeping proteins (ATPase, RNA-polymerase and molecular chaperone DnaK). Locus tags are shown without “Tph_” prefix (e.g., Tph_c21360 is shown as c21360). Data were obtained from triplicate measurements. (TIF) [file pone.0336914.s018.tif]
