# Supplementary material for: Amino acid metabolism of the thermophilic acetogen Thermacetogenium phaeum
Source: PLoS One. 2025 Dec 3;20(12):e0336914. doi: 10.1371/journal.pone.0336914 (PMC12674515; doi:10.1371/journal.pone.0336914)
Supplement: S1 Table — (PDF) [file pone.0336914.s019.pdf]

**Table S1. Specific enzyme activities of glycine cleavage system measured in photometric enzyme assays**

| | electron carrier | soluble fraction (U/mg) | membrane fraction (U/mg) |
|---|---|---|---|
| glycine cleavage system | $NAD^+$ | 0.001±0.00092 | -0.00038±0.00094 |
